# Differential transcriptional networks associated with key phases of ingrowth wall construction in *trans*-differentiating epidermal transfer cells of *Vicia faba* cotyledons

**DOI:** 10.1186/s12870-015-0486-5

**Published:** 2015-04-16

**Authors:** Hui-Ming Zhang, Simon Wheeler, Xue Xia, Ruslana Radchuk, Hans Weber, Christina E Offler, John W Patrick

**Affiliations:** School of Environmental and Life Sciences, The University of Newcastle, Callaghan, NSW 2308, Australia; Leibniz Institute of Plant Genetics and Crop Plant Research (IPK), Corrensstrasse 3, D-06466, Gatersleben, Germany

**Keywords:** Transfer cell, Transcriptome, Cell wall construction, Uniform wall, Wall ingrowth, Transporters, Seed development, *Vicia faba*

## Abstract

**Background:**

Transfer cells are characterized by intricate ingrowth walls, comprising an uniform wall upon which wall ingrowths are deposited. The ingrowth wall forms a scaffold to support an amplified plasma membrane surface area enriched in membrane transporters that collectively confers transfer cells with an enhanced capacity for membrane transport at bottlenecks for apo-/symplasmic exchange of nutrients. However, the underlying molecular mechanisms regulating polarized construction of the ingrowth wall and membrane transporter profile are poorly understood.

**Results:**

An RNAseq study of an inducible epidermal transfer cell system in cultured *Vicia faba* cotyledons identified transfer cell specific transcriptomes associated with uniform wall and wall ingrowth deposition. All functional groups of genes examined were expressed before and following transition to a transfer cell fate. What changed were the isoform profiles of expressed genes within functional groups. Genes encoding ethylene and Ca^2+^ signal generation and transduction pathways were enriched during uniform wall construction. Auxin-and reactive oxygen species-related genes dominated during wall ingrowth formation and ABA genes were evenly expressed across ingrowth wall construction. Expression of genes encoding kinesins, formins and villins was consistent with reorganization of cytoskeletal components. Uniform wall and wall ingrowth specific expression of exocyst complex components and SNAREs suggested specific patterns of exocytosis while dynamin mediated endocytotic activity was consistent with establishing wall ingrowth loci. Key regulatory genes of biosynthetic pathways for sphingolipids and sterols were expressed across ingrowth wall construction. Transfer cell specific expression of cellulose synthases was absent. Rather xyloglucan, xylan and pectin biosynthetic genes were selectively expressed during uniform wall construction. More striking was expression of genes encoding enzymes for re-modelling/degradation of cellulose, xyloglucans, pectins and callose. Extensins dominated the cohort of expressed wall structural proteins and particularly so across wall ingrowth development. Ion transporters were selectively expressed throughout ingrowth wall development along with organic nitrogen transporters and a large group of ABC transporters. Sugar transporters were less represented.

**Conclusions:**

Pathways regulating signalling and intracellular organization were fine tuned whilst cell wall construction and membrane transporter profiles were altered substantially upon transiting to a transfer cell fate. Each phase of ingrowth wall construction was linked with unique cohorts of expressed genes.

**Electronic supplementary material:**

The online version of this article (doi:10.1186/s12870-015-0486-5) contains supplementary material, which is available to authorized users.

## Background

Transfer cells (TCs) *trans*-differentiate from a range of existing cell types belonging to the major tissue systems of dermal (e.g., epidermal cells), ground (e.g., endosperm cells; root cortical parenchyma cells) and vascular (phloem and xylem parenchyma cells; companion cells – [1]). Once TC development is completed, a 10- to 20-fold amplification of their plasma membrane surface area, containing high densities of solute transporters, confer these cells with an extraordinarily high capacity for nutrient exchange between apo- and symplasmic compartments located at bottlenecks for long-distance transport of nutrients throughout the plant body [1]. An intricate invaginated complex of cell wall ingrowths provides structural scaffolding on which the amplified plasma membrane is arrayed to enhance nutrient flows. The tight coupling of this structure/function relationship is graphically illustrated by compromised seed filling of mutants in which TCs, located at the maternal/filial interfaces of both eudicots and monocots, exhibit an attenuated construction of their cell wall ingrowth complex [2,3]. Since a number of major crop species, including cereals and grain legumes, contain TCs positioned at the maternal/filial interfaces of their developing seeds [1], the shrivelled seed phenotype exhibited by TC mutants underscores the important role these cells play in determining crop yields.

The cell wall ingrowth complex is organized into one of two architectural types – flange or reticulate [1]. Flange ingrowths form as ribs or bands of wall material while the more commonly occurring reticulate wall ingrowths arise as numerous wall papillae that develop at right angles to the original wall. The extent of their reticulation varies from cylindrical papillae alone to ones in which papillae branch and fuse to generate fenestrated wall layers [4]. Immediately preceding construction of reticulate, but not flange wall ingrowths [5], a structurally distinctive wall-layer, the so-called uniform wall, is rapidly laid down over the pre-existing primary wall of the *trans*-differentiating cell to a thickness that can extend up to 50% of that of the pre-existing primary wall [6]. Collectively, the uniform wall and reticulate wall ingrowths form the ingrowth wall [1].

Despite the central role the ingrowth wall, and particularly its wall ingrowth component, plays in underpinning transport function of TCs, little is known about the mechanisms responsible for inducing and then orchestrating its construction. Transcriptional analyses of cells committed to a developmental pathway leading to forming a TC-morphology have identified up-regulated expression of genes encoding components of signalling pathways for abscisic acid, auxin, ethylene, reactive oxygen species (ROS) and Ca^2+^ in developing seeds [1,7-9] and for auxin and ethylene in giant cells induced by nematode infection [10]. Events downstream from the inductive signals largely have been identified by transcriptome analyses of developing cereal seeds and, in particular, for flange wall ingrowths formed in basal endosperm TCs (BETCs) of maize [11] and barley [8,12]. A more limited analysis has been reported for the formation of reticulate wall ingrowths in eudicots [10,13].

While there are undoubtedly some shared features, given the disparate architectures of flange and reticulate wall ingrowths, we hypothesize that signalling pathways, cell wall biosynthesis and delivery of polysaccharides to the wall matrix will have features that are peculiar to each architectural type of ingrowth wall. This characteristic likely extends to distinctive elements responsible for constructing the uniform wall and wall ingrowth papillae. To this end, we used *Vicia faba* cotyledons, in which on transfer to culture, their adaxial epidermal cells spontaneously undergo *trans*-differentiation to a TC-morphology [1]. This experimental system provided the opportunity to undertake a transcriptome analysis of TC-specific gene networks [1]. In addition, because of the temporal sequence for the deposition of the uniform wall followed by wall ingrowth papillae, gene cohorts associated with these two developmental events could be distinguished. Hereafter, wall ingrowth papillae, which represent the initial stage of wall ingrowth construction [4], are referred to as wall ingrowths.

## Methods

### Plant growth conditions, cotyledon culture and collection of tissue samples for sequencing

*V. faba* L. (cv. Fiord) plants were raised under controlled environmental conditions [13]. Cotyledons of harvested pods were surgically excised and cultured aseptically on liquid MS medium for specified times (see below) before being fixed in 75% ethanol and 25% acetic acid for 1 h at 4°C. Peels of the adaxial epidermis and blocks of storage parenchyma cells (2 × 2 × 1 mm) were surgically removed from each fixed cotyledon. Collected tissues were immediately snap-frozen in liquid nitrogen and stored at -80°C until used for RNA extraction.

**Figure 1:**
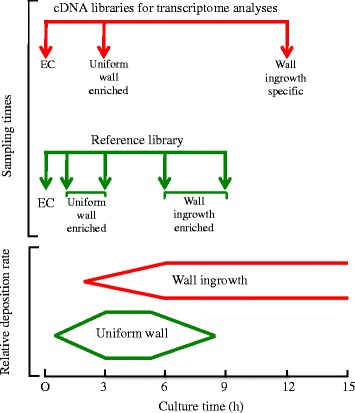
Tissue sampling times (vertical arrows) informed by the sequential temporal deposition of the uniform wall followed by wall ingrowth papillae. Note that across the period sampled, wall ingrowth papillae continue to be deposited and have not commenced to form the first fenestrated layer [4]. EC, epidermal cell.

Selection of times to obtain tissue samples from cultured cotyledons was based on the temporal pattern of uniform wall deposition preceding that of wall ingrowths (Figure 1 and, for more information, see Additional file 1). To this end, for the reference library, a representative balance of genes induced to regulate uniform wall and wall ingrowth construction was obtained by collecting 6 mg of epidermal peels from each of freshly harvested cotyledons (0 h – reference to identify genes induced/switched off during *trans*-differentiation) and cotyledons cultured for 1 and 3 h (dominated by expression of genes regulating uniform wall formation), 6 and 9 h (dominated by expression of genes regulating deposition of wall ingrowths as uniform wall formation ceased). To identify expression of TC-specific genes regulating uniform wall and wall ingrowth construction, epidermal peels (15 mg) and storage parenchyma tissue (30 mg) were sampled from replicate batches of freshly harvested cotyledons and cotyledons cultured for 3 h (dominated by uniform wall construction) and 12 h (wall ingrowth construction alone and for additional information see Additional file 1 and Results).

To verify that each sampled population of cultured cotyledons was *trans*-differentiating, percentages of epidermal cells forming wall ingrowths of a sub-set of cotyledons at 12 h of culture were scored (see Additional file 1 for Methods).

### RNA isolation, cDNA-library construction and Illumina sequencing

Total RNA was extracted using Qiagen (USA) RNeasy plant mini kits. Contaminating genomic DNA was removed using DNase I.

For preparation of an epidermal-specific TC transcriptome reference library, 1 μg of total RNA was aliquoted from the total RNA extracts of each specified harvested time point. The RNA aliquots were combined to generate a temporally mixed RNA library (5 μg of total RNA). To identify genes responsible for uniform wall and wall ingrowth formation, 2 μg aliquots of total RNA were sampled from each biological replicate of epidermal and storage parenchyma RNA extracts.

Total RNA quality was verified by determining the integrity of the 25S and 18S RNA with an Agilent 2100 Bioanalyzer (Agilent, USA and see Additional file 2). The cDNA libraries were prepared from poly-A mRNA isolated from 1 μg of total RNA using a TruSeq® RNA v2 sample prep kit (Illumina, USA) according to manufacturer’s instructions*.* cDNA quality was evaluated by determining size and purity using an Agilent 2100 bioanalyzer (see Additional file 3). cDNA fragments, ranging from 100 – 700 bp, were selected by agarose gel purification. For the reference library, selected cDNA fragments were 100 bp pair-end sequenced in a single lane on an Illumina HiSeq 2000 platform (Australian Genome Research Facility, Melbourne).

To characterize gene cohorts linked with uniform wall and wall ingrowth deposition, only biological replicates with the anticipated 70% (or more) of their epidermal cells at 12 h of cotyledon culture containing detectable wall ingrowths [14] were processed for Illumina sequencing. In three compliant biological replicates for each specified harvest time, cDNA libraries (18 in total) were prepared from total RNA extracts of epidermal peels and storage parenchyma tissues as indicated above (and see Additional files 2 and 3 for RNA and cDNA quality checks). cDNA fragments were purified separately from each sample and indexed with unique nucleic acid identifiers (Illumina TruSeq V2 index sequence). The indexed cDNA libraries were diluted to an average concentration of 10 nM and pooled in equal volumes (10 μL of each library) to generate the final mixed cDNA pool for sequencing. The pool was then 100 bp pair-end sequenced in two lanes on an Illumina HiSeq 2000 platform (Australian Genome Research Facility, Melbourne).

Using Illumina CASAVA pipeline version 1.8.2, raw reads were trimmed with adaptor filtering and a read length cut-off of 50%. Thereafter, filtered reads with over 20% of their nucleotides having a Q score < 20 (probability of sequencing error > 0.01) or their sequences having a N reading over 5% were removed.

### *De novo* assembly, annotation and GO classification of transcriptome library

Filtered reads from the cDNA library were assembled *de novo* into contigs by Beijing Genomics Institution (BGI, Shenzhen, China) using Trinity software release-20130225 [15] with a k-mer of 25 and a minimum k-mer threshold abundance of 2 (min_kmer_cov 2). The reads were then mapped back to contigs to assemble unigenes using Trinity Butterfly that filtered out transcriptional artifacts, misassembled transcripts and poorly supported transcripts. Potential redundant sequences were grouped, using the TGI Clustering Tool set with a minimum 40% sequence overlap and over 80% sequence identity.

Assembled unigene sequences were annotated by alignment to the following publically available databases: NCBI nr (non-redundant, see http://www.ncbi.nlm.nih.gov/refseq/); Swissprot (http://www.uniprot.org/); KEGG (http://www.genome.jp/kegg/); COG (https://www.ncbi.nlm.nih.gov/COG/), using BLASTX with an e-value threshold of 1e^-5^. Unigenes with no hit in BLASTX were predicted using ESTScan.

### Mapping reads to the reference library, determining differentially expressed genes and GO-enrichment analysis

Reads sequenced by the Illumina HiSeq 2000 platform from mRNA extracts of cotyledon epidermal peels and storage parenchyma tissues were analyzed by BGI. Raw reads were filtered as described for the reference transcriptome library. Clean reads were aligned to the reference sequences (generated as described above) using the SOAPallgner/SOAP2 pipeline. No more than 5 mismatches per read were allowed to ensure high quality alignment. Sequence coverage of unigenes in each sample was calculated. During alignment, SOAPallgner/SOAP2 reported the number of mapped reads per kilo base per million reads (RPKM) as a measure of transcript abundance of each unigene. Transcripts with RPKMs < 0.45 were considered not to be expressed and were removed from the data sets.

Differentially expressed genes (DEGs) were determined using a computational algorithm based on digital gene expression profiles [16] to perform pairwise differential expression analysis. Multiple testing of the comparisons was corrected using the FDR method [17]. Sequences with a corrected FDR P value of no more than 0.05 were selected. After this correction, genes exhibiting a differential expression of two-fold or more (P < 0.05) were identified as DEGs. Subcellular localization of selected proteins encoded by unigenes was predicted using the WoLF PSORT algorithm.

### Quantitative RT-PCR validation

A collection of 15 unigenes with different expression patterns (see Additional files 4 and 5 for expression pattern information) was selected. cDNA was converted from the same RNA samples sent for Illumina sequencing using QuantiTect Reverse Transcription Kit (Qiagen, USA). Primers were designed using Primer 3 plus (Whitehead Institute for Biomedical Research, USA) and synthesized by Sigma-Aldrich Australia (see Additional file 6 for primer sequences). For each qRT-PCR reaction a 15 μL system containing 7.5 μL SYBR Green master mix (Qiagen, USA), 0.375 μL of forward and reverse primers (10 μM), 1.75 μL of nuclease free H_2_O and 5 μL cDNA was set up. The following PCR cycle was used: 95°C for 5 min, 95°C for 15 s, 60°C for 20s, 72°C for 30 s; steps 2 to 4 were repeated 50 times. High-resolution melting curves (72–95°C) following the final PCR cycle checked the specificity of the PCR products. For each cDNA sample, technical duplicates in each of three biological replicates were tested. Four housekeeping gene candidates were assessed using GeNorm. Relative expression levels of each unigene were determined using the two standard curves method.

## Results

### *De novo* assembled transcriptome library for ingrowth wall deposition

Sequencing of reference library cDNA fragments on an Illumina HiSeq 2000 platform generated 181,419,640 pair-end reads (100 bp). After filtering raw reads, 126,200,279 high-quality reads, in which 97% of nucleotides have a Phred quality score of ≥ Q20 level (error probability ≤ 0.01) (Table 1) were *de novo* assembled into 131,279 contigs (>200 bp) with a N50 value of 980 bp and an average length of 423 bp. Clustering yielded 74,659 unigenes with 33,902 consensus sequences grouped into 11,083 distinct clusters and 41,567 singletons (Table 1). This unigene population had a N50 value of 1723 bp and an average length of 1076 bp. High integrity and accuracy of the assembly was indicated by 85.2% of filtered reads mapping to the assembled transcriptome with 41.4% of reads uniquely mapping to the transcriptome (Table 1). The uniquely mapped reads had an average coverage depth of 8.4 (12,620 bp per mRNA).

**Table 1:** **Raw, filtered and mapped reads used for the** ***de novo*** **assembly of the reference transcriptome library for adaxial epidermal cells of freshly harvested and cultured** ***V. faba*** **cotyledons undergoing** ***trans*** **-differentiation to a TC morphology**

**Category**	**Number**	**Percentage (compared with total number of reads)**
Raw reads	181,419,640	100%
Clean reads	126,200,279	69.56%
Mapped reads	107,093,666	59.03%
Uniquely-mapped reads	44,339,744	24.44%
Multiple-mapped reads	62,753,921	34.59%

BLASTX searches of publically available protein databases, using the putative unigenes as query sequences, yielded matches for 68.9% of the unigenes. BLASTX-derived alignments predicted sequence orientation for 43,910 of the unigenes and identified 43,703 open reading frames. Taxonomic distribution of the annotated unigenes by Blast2GO demonstrated that over 85% unigenes had their best hits (smallest e value) with their homologs in other legume species (see Additional file 7). The raw reads and assembled unigene sequences are available at European Nucleotide Archive, accession number: PRJEB8906.

### Global gene expression patterns during early phases of *trans*-differentiation to a TC-morphology

Total RNA was extracted from epidermal peels and storage tissue blocks at 0, 3 and 12 h of cotyledon culture and processed for RNAseq on an Illumina platform (see Methods). Between 19 – 37 million clean reads were generated for each biological replicate (see Additional file 8). An average of 85% of the clean reads mapped to expressed unigenes. Of these reads, 60% mapped with a perfect match and 40% with a mismatch less than 5 bp (Additional file 8). Total numbers of unigenes, detected within epidermal and storage parenchyma cells across cotyledon culture were temporally stable (Additional file 9). Some 70% of the unigenes exhibited expression levels > 0.45 RPKM within a cell type and these were dominated by unigenes with expression levels ranging from 0.5 to 10 RPKM (80%).

**Figure 2:**
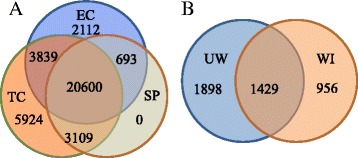
Numbers of unigenes expressed preferentially in, or shared between, (**A**) cell types and (**B**) uniform wall and wall ingrowth formation occurring within adaxial epidermal cells of cultured *V. faba* cotyledons. Only unigenes with a RPKM value > 0.45 in all three biological replicates were defined as ‘expressed’ in one or more cell types/phases of ingrowth wall deposition. For (**B**), only unigenes with a coverage of > 70% were listed for further analysis. EC- adaxial epidermal cell; TC – epidermal TC *trans*-differentiated from adaxial epidermal cell; SP- storage parenchyma cell; UW - uniform wall; WI- wall ingrowth.

An estimated 27,244 unigenes were expressed in adaxial epidermal cells of developing *V. faba* cotyledons (Figure 2A). Of these epidermal cell unigenes, 21,293 were shared with the underlying storage parenchyma cells leaving 5,951 unigenes that were epidermal cell specific (Figure 2A). Upon transiting to a TC fate, expression of 2,805 unigenes in the precursor epidermal cells was switched off (Figure 2A). The combined transcriptomes of *trans*-differentiating TCs at 3 and 12 h of cotyledon culture totalling 33,472 unigenes approximated those detected by RNAseq analyses of differentiating BETCs in barley grains (42,086 – [8]) and of nematode giant TCs in rice roots (42,756 unigenes at 7 days post infection; 41,179 genes at 14 days post infection – [18]). Seventy-three % of the epidermal TC transcriptome was represented by ongoing expression of genes by their precursor epidermal cells whilst 27% were induced (Figure 2A). The percentage of induced genes was comparable to the proportion of up-regulated genes in nematode giant TCs [18]. Of the induced genes, 66% were TC specific and the remainder was shared with the underlying storage parenchyma cells (Figure 2A). Ten % of the epidermal cell genes were silenced in transiting to an epidermal TC identity and these were replaced 3-fold by induced genes (Figure 2A). TC specific genes were inspected manually to remove duplicates and those with < 70% coverage to yield a total of 4,283 unigenes (Figure 2B).

### Transcriptome networks specifically expressed during uniform wall or wall ingrowth formation

The temporal sequence of uniform wall followed by wall ingrowth deposition (Figure 1; Additional file 1) provided the opportunity to separate the TC-specific cohort of expressed genes into groups linked with the two phases of ingrowth wall construction and those shared between these phases as follows. The latter were identified by manual inspection of the 3- and 12-h gene expression profiles (Figure 2B). The remaining expressed genes were then specifically linked with either uniform wall or wall ingrowth formation and these were identified on the following grounds. At 12 h of cotyledon culture, uniform wall construction had ceased and TC-specific gene expression is solely committed to deposition of wall ingrowths (Figure 1; Additional file 1). Thus the expression profile at 12 h contained transcripts specifically related to constructing wall ingrowths alone (Figure 2B). In contrast, at 3 h of cotyledon culture, uniform wall construction is proceeding rapidly concurrent with a smaller population of cells starting to deposit wall ingrowths (Figure 1, Additional file 1). Hence at 3 h of culture, genes involved in uniform wall and wall ingrowth construction would be co-expressed. However, those exclusively directing deposition of wall ingrowths would be expressed at relatively lower levels. Thus subtracting the 12-h (wall ingrowths alone) from the 3-h (uniform wall plus wall ingrowths) gene expression profile identified those genes specifically expressed during uniform wall construction (Figure 2B).

The above exercise demonstrated that of the TC-specific expressed genes, 66% were selectively linked with uniform wall (44%) or wall ingrowth (22%) formation with the remainder (34%) being expressed throughout ingrowth wall formation (Figure 2B and see Additional file 4 for their RPKM gene expression values). This latter cohort of genes was categorised according to their relative expression levels in the two phases of ingrowth wall formation, that is, no change and up-regulated during uniform wall or wall ingrowth deposition. Genes whose expression levels did not change accounted for 60% of the common cohort (Table 2).

**Table 2:** **Numbers of annotated expressed genes ascribed to specified functional categories for genes switched off in epidermal cells transiting to a TC fate and for those specifically expressed in epidermal cells undergoing** ***trans*** **-differentiation to a TC morphology**

**Functional category**	**Epidermal switched-off genes**	**Transfer cell specific expressed genes**				
		**UW/WI no change**	**UW up-regulated**	**UW specific**	**WI up-regulated**	**WI specific**
**Total number of expressed genes**	1381	851	366	1898	214	958
**Total number of annotated genes**	468	364	217	647	138	407
**DNA synthesis and modelling**	17	8	1	13	2	13
**Transcription/translation**	32	27	11	31	3	19
**Signalling**						
Receptor kinase/kinase	15	17	20	37	8	16
Hormonal	4	6	6	13	8	14
ROS/Ca^2+^	1	4	9	6	1	6
**Intracellular organization**						
Cytoskeleton/vesicle trafficking	10	6	2	11	1	7
Membrane microdomains	0	3	5	3	1	7
**Cell wall enzymes and structural proteins**						
Cellulose	3	0	1	3	1	1
Matrix polysaccharides	9	4	6	14	3	7
Structural proteins	2	3	3	3	5	5
**Membrane transporters**	14	13	9	12	1	13
**Defense**	4	10	21	12	1	6
**Flavonoid synthesis and compartmentation**	5	6	4	3	4	8

TC specific genes separated into those genes expressed throughout uniform wall (UW) and wall ingrowth (WI) formation and those that are specific to each of these wall-building phases. Genes expressed throughout ingrowth wall formation are separated into groups depending on their differential expression patterns of no change, up-regulated during UW or WI formation (for more details, see Results).

BlastX searches, at a stringency of < e^-5^, established that 41% of the TC-specific genes and 34% of epidermal cell genes switched off shared homology with known genes listed in public databases (Table 2). The 59/66 % of un-known transcripts equates with a similar value reported for the transcriptome of developing barley BETCs [12].

Table 2 provides a summary of the changes in expression of selected functional categories of TC-specific genes that are known to be central to TC development and function. For all categories, numbers of epidermal cell genes switched off in transiting to a TC fate were replaced by increased numbers of TC-specific transcripts between 3- to 12-fold. Amongst these, expression of transcription/translation, receptor kinase/kinases, cytoskeleton/vesicle trafficking, matrix polysaccharides and defense genes contributed to the two-fold greater number of genes expressed during uniform wall compared to wall ingrowth construction (Table 2; Figure 2B). In addition, a portion of uniform wall associated expressed genes were linked with a co-occurring burst in cell division/expansion (Additional file 10). Genes encoding the entire flavonol pathway were switched on during TC development (Table 2).

A more detailed analysis was undertaken of the temporal expression of TC-specific genes contained in functional groups that are at the heart of regulating development of the TC’s structural specialization of a polarized ingrowth wall that realises their functional capacity to support high rates of nutrient transport across their plasma membranes [1]. These categories included hormonal and ROS/Ca^2+^ signal generation and their signalling pathways, cytoskeleton/vesicle trafficking and lipid biosynthetic enzymes related to microdomain formation, cell wall biosynthetic and remodelling enzymes along with cell wall structural proteins and membrane transporters (see Additional files 11, 12, 13 and 14 inclusive). These data are supplemented with estimates of their annotation and relative expression levels at specified stages of ingrowth wall construction along with noting whether their homologues have been detected in transcriptomes of BETCs and nematode giant TCs (Additional file 5). Temporal patterns of expression of selected genes from the targeted functional categories determined by qRT-PCR were consistent with those derived from Illumina sequencing (Additional file 15), suggesting that the Illumina data are relatively reliable (Table 2, Additional files 4, 5, 11, 12, 13, and 14).

## Discussion

**Figure 3:**
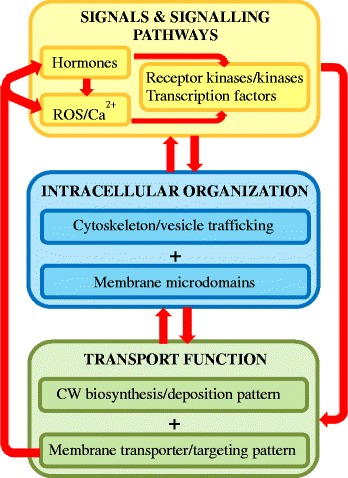
Regulatory interrelationships (red arrows) between the functional categories of expressed genes that contribute to developing a functional transfer cell.

The ability to obtain peels of cotyledon epidermal cells permitted cell-specific transcriptomes of these cells to be identified as they *trans*-differentiated to a TC-morphology [13]. In addition, developmental stage-specific transcriptomes were isolated (Figure 2, Additional files 5, 11, 12, 13, and 14) by taking advantage of the temporal disjunction between uniform wall and wall ingrowth construction (Figure 1, Additional file 1). We interrogated these TC-specific transcriptomes to discover genes encoding proteins of functional categories considered to contribute to developing a transport capable TC (Figure 3 and see Additional files 5, 11, 12, 13, and 14). Transcriptional cascades are initiated by auxin and ethylene [13,19,20] along with ROS and Ca^2+^ serving as secondary messengers and positional signals [6,9]. The substantial numbers of expressed receptor kinases/kinases and transcription factors, point to these forming a significant component of the signalling network (Table 2 and see [12]). Re-organization of the cytoskeleton and vesicle trafficking together with membrane microdomains, under control of the inductive signalling network, would be expected to direct targeted delivery of cargos containing cell wall biosynthetic enzymes and cell wall components to construct the polarized uniform wall and localized wall ingrowths (Figure 3 and Additional file 1). The latter provide a scaffold to support an amplified plasma membrane surface area to which membrane transporters are targeted through a polarized endomembrane secretory system to confer enhanced TC transport function (Figure 3). In addition, targeted positioning of transporters is responsible for generating the polarized extracellular ROS [6] and cytosolic Ca^2+^ [9] signals and possibly altering positioning of auxin transporters that may contribute to generating the auxin maximum ([13]; Figure 3). The following discussion focuses on expressed genes belonging to these three functional categories namely, signals and signalling pathways, intracellular organization and transport function (Figure 3). The discussion starts with seeking out candidates responsible for generating the developmental signals and downstream signalling pathways. In all cases, statements regarding gene identity and function should be considered as putative.

### Signals and signalling pathways

Hormonal signals, acting in series with ROS and Ca^2+^ [1], likely activate expression of receptor kinases/kinases and transcription factors to induce TC *trans*-differentiation (Figure 3).

#### Auxin, ethylene and abscisic acid form a complex of primary signals putatively regulating TC development

Consistent with previous findings for epidermal TCs [13,19] and those reported for nematode giant TCs (10), induction of *trans*-differentiation was accompanied by changes in auxin activity (as determined by expression of auxin-induced genes) increasing five-fold between uniform wall and wall ingrowth formation driven by an auxin maxima generated by enhanced auxin biosynthesis and disrupted polar transport (Additional file 11). For instance, auxin biosynthesis could be elevated by expression of *indole-3-acetate O-methyltransferase IAA*, that catalyzes synthesis of a biologically more active IAA, methyl-indole-3-acetic acid [21] and *indole-3-glycerol phosphate synthase* that starts the tryptophan independent IAA biosynthetic pathway [22]. Polar auxin transport was likely disrupted by de-localized distribution of expressed auxin efflux and influx carriers by co-expression of *KAN* and *HD-ZIPIII* [23]. As a point of distinction between mechanisms regulating flange and reticulate wall ingrowths, in contrast to the latter [10,13,19], differentially expressed auxin-related genes were not detected in developing BETCs [8,11].

Initiation of epidermal TC development is linked with a substantive burst in ethylene biosynthesis, regulated by an auxin-induced expression of *ACC synthases* [19], driving expression of *ethylene response factor*s (*ERF*s) [19] and particularly so for uniform wall formation (Additional file 11). For example, *ERF2.6* and *SHINE* expression could invoke cell wall biosynthesis through respectively up-regulating callose and cellulose biosynthesis (Additional file 13 and see [24,25]). Strong expression of ethylene signalling genes during uniform wall formation corresponded with that reported for early phases of wall ingrowth construction in barley BETCs [8] and nematode giant TCs [10].

A switch to a specific ABA signalling pathway throughout ingrowth wall formation was suggested by expression of *PYL6*, an ABA receptor [26], *LEC1*, tightly connected with ABA signalling regulating *AB13* and *AB15* [27] and *ERF RAP2.6* that acts through an AB14-mediated signalling pathway ([28]; Additional file 11). ABA also acts during wall ingrowth formation (Additional file 11). ABA action throughout ingrowth wall formation in epidermal TCs contrasts with that for barley BETCs where ABA induces a maturation program once wall ingrowth deposition ceases [8].

Three gibberellin biosynthetic genes with the biological activities of their products being modulated by two *gibberellin 2-oxidases* and *Short Internode Related* during uniform wall formation are expressed (Additional file 11). These gibberellins likely regulate cell division and expansion (Additional file 10 and see [29]). An absence of gibberellin activity during wall ingrowth construction was suggested by co-expression of *Short Internode Related* and *gibberellin 2-beta-dioxygenase 8-like* (Additional file 11; [29]). Residual cytokinin levels in the developing epidermal TCs would be depleted through degradation by the expressed *cytokinin dehydrogenase* [30] and by conjugation to an inactive form catalyzed by the five cytokinin-O-glucosyltransferases ([31]; Additional file 11). No components of the salicylic acid or brassinosteroid signalling pathways were detected in the TC-specific transcriptome (Additional files 4 and 11) indicating that these pathways likely did not play a role in *trans*-differentiation to a TC state.

#### Reactive oxygen species and Ca^2+^ serve as secondary messengers directing ingrowth wall construction

Extracellular ROS, produced by ethylene-induced respiratory burst oxidases (rbohs), regulate biosynthesis and polarized deposition of the uniform wall in epidermal TCs [6,32]. Similarly, rboh expression co-occurs with wall ingrowth formation in barley BETCs [8]. In contrast, no differentially expressed *rboh*s were detected in nematode giant TCs [10]. Interestingly, ROS-related gene expression was dominated by those involved in extra- and intracellular ROS homeostasis (Additional file 11) suggesting that ingrowth wall formation depends upon a spatio/temporal fine-tuning of ROS. For instance, antioxidant capacity could be down regulated by ascorbic acid oxidation catalyzed by an extracellular L-ascorbate oxidase ([33]; Additional file 11) whilst concurrent ROS degradation by peroxidation [32] could be mediated by carboxymethylenebutenolidase-like protein (Additional file 11), secreted to the cell wall [34]. In contrast to epidermal TCs, differentially expressed peroxidases were not detected in barley BETCs [8] and were strongly down-regulated in nematode giant TCs [10].

Loci at which wall ingrowths are deposited are defined by a cytosolic Ca^2+^ signal spatially organized into narrow plumes shaped by co-operative activities of plasma membrane Ca^2+^-permeable channels organized into clusters surrounded by Ca^2+^-ATPases [9]. However, unlike the unknown DHP receptor Ca^2+^-permeable channels responsible for generating the Ca^2+^ signal [9], ion conductances of the expressed SKOR, annexin and cyclic nucleotide gated channels are not inhibited by verapamil or nifedipine (e.g. [35]). This, together with channel expression coinciding with uniform wall construction (Additional file 11), points to an additional role for Ca^2+^ signalling in transiting precursor epidermal cells to a TC fate. A conclusion that accommodates Ca^2+^ signalling genes being expressed in both flange [8] and reticulate forms [18,36] of developing TCs. Ca^2+^ signalling would appear to be mediated through Ca^2+^-dependent protein kinases during uniform wall formation while a calmodulin signalling route could operate when wall ingrowths are being deposited as suggested by expression of three IQ-domain containing proteins (Additional file 11) that bind calmodulins to facilitate their function.

#### Receptor kinases/kinases represent a significant component of the signalling network

TC-specific receptor kinases/kinases represented a major component of the signalling networks (59%) expressed within the epidermal TCs (Table 2). While their functions are unknown, similar to nematode giant TCs [10], members of a two component signalling system present in barley BETCs [12], was not detected in the epidermal TC cohort of receptor kinases/kinases. Thus, the presence/absence of a two component signalling system could underpin the distinction between flange [12] and reticulate ([10]; this study) TC wall architectures.

### Intracellular organization

Polarized accumulation of extracellular ROS regulating uniform wall synthesis [6] and cytosolic Ca^2+^-plumes determining loci for wall ingrowth formation [9] respectively depend upon a finely-tuned intracellular distribution of rbohs and Ca^2+^-permeable channels. This requirement is undoubtedly met through modifying cytoskeleton and vesicle trafficking combined with re-configuring microdomains in the plasma membrane abutting sites of ingrowth wall deposition (Figure 3).

#### TC-specific expression of cytoskeleton and vesicle trafficking confers TC polarity

Microtubule and actin-associated genes switched off on transiting to a TC fate were predominantly linked to cell division (Additional file 12) that declined rapidly from three to six hours (Additional file 10). Consistent with polarized cell wall deposition [4], TC- specific genes indicative of remodelling cytoskeletal elements [37] were expressed either across ingrowth wall deposition (*kinesin motor domain* and *formin-like protein*) or only during one phase of the process (Additional file 12). Uniform wall specific gene expression (Additional file 12) indicated substantial polymerization/reorganization of actin filaments (*actin* and *actin-97-like*) and polarization of transport vesicles and organelles (*Myosin X1*) [38] consistent with ROS-regulated polarization of the uniform wall [6]. Wall ingrowth specific expression of *65-kDa microtubule-associated protein 3-like* and *Villin-4* respectively suggests establishing a stable microtubule [39] and actin [40] network to orchestrate deposition of wall ingrowths at loci. In root hair growth of Arabidopsis, *AtVLN4* regulated actin organization is Ca^2+^-dependent [41] pointing to an interaction between actin and Ca^2+^plumes that define wall ingrowth loci [9].

Rapid polarized/localized cell wall deposition during formation of the ingrowth wall (Additional file 1) is dependent upon exo/endocytotic activity [13]. Regulation of cycling of ER-derived proteins through assembly and directed transport of coat protein complex II (*GTP-binding protein SAR1A* and *ER-derived vesicle protein ERV14*) and ARFs (*ADP-ribosylation factor GTPase-activating proteins*) across ingrowth wall deposition is consistent with asymmetrical vesicle trafficking to effect polarity [42,43]. Expression of uniform wall- and wall ingrowth-specific *exocyst complex components* and v- and t-SNARES (*Vesicle-associated protein 2-1-like, Vesicle-associated membrane protein* and two*, Syntaxin -112* genes), to direct targeted vesicle delivery to the plasma membrane [44], confirms the specialized nature of each phase of ingrowth wall deposition. Two of four genes encoding proteins involved in membrane trafficking (*Dynamin –related protein 1C-like* and *Dynamin-2B-like*) are potential regulators of localized endocytosis supporting wall ingrowth deposition. These two dynamin genes represent two subfamilies of DRPs that interact and assemble with clathrin at discrete foci in the plasma membrane of Arabidopsis cells to regulate endocytosis [45] and cellulose deposition [46]. This profile of cytoskeleton and vesicle trafficking genes is comparable to that reported for BETCs [8] and nematode giant TCs [18].

#### Formation of membrane microdomains may sub-compartmentalize the plasma membrane polar domain

Sphingolipids and sterols form major components of membrane microdomains [47]. Indeed, genes encoding enzymes in their biosynthetic pathways are expressed during development of flange wall ingrowths in barley BETCs [8]. Sphingolipids consist of a polar head group linked to an amino alcohol long-chain base (LCB) with the amine group acylated with a fatty acid (ceramide) with C4 of LCB being (trihydroxy) or not being (dihydoxy) hydroxylated. *Bax inhibitor 1-like* (Additional file 12) post-translationally activates the C4 hydrolase [48] thus promoting flux through this route for acylation by very long-chain fatty acids (VLCFA; [49]). Significantly, synthesis of VLCFAs may be enhanced by expression of four *3-ketoacyl-CoA synthase* isoforms, a *elongation of fatty acid protein* and a *long-chain-fatty-acid-CoA ligase* (Additional file 12) that form part of the elongase complex [50]. An increased precursor flow into the VLCFA pathway is supported by up-regulated expression of *biotin carboxyl carrier protein of acetyl-CoA carboxylase* (Additional file 12), a sub-component of acetyl-CoA carboxylase catalyzing the first carboxylation step in, and rate limiting of, *de novo* fatty acid synthesis [51]. Thereafter, *bax inhibitor 1-like* may also increase activities of fatty acid hydroxylases and desaturases that further modify the ceramides [48] before synthesis of the final sphingolipid products of glucosylceramides or glycosyl inositolphosphoceramides [49]. Uniform wall specific expression of an *oxysterol-binding protein-related protein* and a *remorin* (Additional file 12) respectively point to vesicle trafficking of sterols [52] and their enrichment in membrane microdomains [47]. Wall ingrowth deposition is linked with expression of putative sterol biosynthetic genes (Additional file 12) such as two *epoxide hydrolases* [53] and *squalene epoxidase* [54].

### TC transport function

Expression of cell wall synthesizing and re-modelling enzymes underpins construction of the ingrowth wall that supports the amplified plasma membrane in which are embedded high densities of membrane transporters to collectively confer an enhanced transport function (Figure 3).

#### Expression of cell wall biosynthesis and re-modelling genes construct the ingrowth wall

Cellulose is present in the uniform wall and the inner, electron dense region, of wall ingrowths of epidermal TCs [55] but cellulose biosynthesis appeared not to depend on expression of TC-specific *CesA*s that were switched off and not replaced as the epidermal cells transited to a TC fate (Additional file 13). Rather, cellulose deposition could be regulated post-translationally (Additional file 13). Thus during uniform wall formation, *SHINE* [56] and a *GPI-anchored protein* [57] could regulate CesA activity with the extruded cellulose microfibrils being re-modelled by two extracellular *endoglucanases* [58]. Cellulose provides a structural scaffold to form wall ingrowths at right angles to the uniform wall [59]. Here, an *acid endochitinase-like protein,* known to associate with cellulose synthase complexes [25] and an extracellular *beta-glucosidase*, may form part of the machinery redirecting extrusion of cellulose microfibrils (Additional file 13). Consistent with these observations, CesA expression is down-regulated in nematode giant TCs [36] while in maize and barley BETCs, CesA expression is up-regulated during later stages of TC development [8,11].

In contrast to cellulose biosynthesis, there was a substantial TC-specific induction of genes encoding enzymes responsible for synthesizing and re-modelling cell wall matrix polysaccharides (Table 2) and, in particular, hemicelluloses (Additional file 13). To this end, a (1-3,1-4) β-D-glucan synthase, was switched off and replaced with enzymes catalyzing xylan biosynthesis (*xylosyltransferases*, *glycotransferase family GT8 protein* – [60]) and re-modelling (*beta-D-xylosidase* – [61]). Xylans in the uniform wall are consistent with their presence in flange wall ingrowths as suggested by expression of a *beta-D-xylosidase* and a *xylosyltransferase* respectively in BETCs of barley [8] and maize [11]. Xyloglucan presence in uniform walls [55] is consistent with expression of xylosyltransferases (biosynthesis - [62]) and endoxylotransglucosylase/hydrolases (chain re-modelling – [63]). Xyloglucan biosynthesis and re-modelling also dominates early developmental stages of barley BETCs [8] and nematode giant TCs [10]. Xyloglucan re-modelling coincides with TC division/expansion as indicated by co-expression of expansins ([8,10,11]; Additional files 10 and 13 in this study). In contrast, expansins expressed during wall ingrowth construction when cell division/expansion has ceased (Additional files 10 and 13), must serve a yet-to-be identified function. One possibility is to free xyloglucan chains from cellulose microfibrils rendering them available for co-operative re-modelling by *alpha-xylosidases* and a *beta-glucosidase* [64] and by two extracellular isoforms of *beta-galactosidase* ([58] – Additional file 13). Overall, uniform wall and wall ingrowth construction was characterized by intense xyloglucan re-modelling.

Pathways leading to pectin biosynthesis and modification altered on epidermal cells transiting to a TC fate (Additional file 13). During uniform wall construction, synthesis of the pectin rhamnogalacturon backbone (RG1) was likely catalyzed by expression of *galactofuronosyl-transferase 13-like* (GAUT13 - Additional file 13) that significantly is essential for polarized pollen tube tip growth [65]. Control over the pectin gel state is conferred by co-expression of a pectin esterase inhibitor modulating catalytic activities of expressed *pectin esterases* (Additional file 13) that catalyze removal of methyl ester groups from homogalacturonan backbones to allow their cross-linking with Ca^2+^ [66]. This accounts for esterified pectins being the predominate type present in the ingrowth wall [55]. Up-regulated and wall ingrowth specific expression of two *beta-galactosidases* could catalyze cleavage of terminal galactosyl residues from RGI [67]. Overall, uniform wall formation included pectin biosynthesis and re-modelling giving way to pectin re-modelling alone for wall ingrowth deposition. Enhanced pectin biosynthesis and re-modelling also characterizes construction of barley BETCs [8] and nematode giant TCs [36].

Callose deposition is a balance between synthesis by callose synthases and degradation by β-1, 3-glucanases. Isoforms of both enzymes were switched off in epidermal cells on transiting to a TC fate and were not replaced during uniform wall formation (Additional file 13). However, *UDP-glucuronosyltransferase 1* (*UGT1*), a callose synthase complex component essential for callose synthesis [68], was expressed during wall ingrowth construction accompanied by two *beta-1, 3-glucanase* isoforms (Additional file 13); an expression pattern consistent with callose being confined to the outer sheath enveloping each wall ingrowth [55]. This temporal profile of callose deposition corresponds with that reported for nematode giant TCs [36] but contrasts with callose synthesis/turnover being restricted to the cell division phase of barley BETC development [69].

Transcripts encoding enzymes of the phenylpropanoid pathway occurred across ingrowth wall formation (Additional file 13). Expressed ABC transporters (Additional file 14) could efflux the monolignol precursors to the cell wall where class III peroxidases (Additional file 11) catalyze their polymerization to lignin [70]. Significantly, the full complement of phenylpropanoid pathway enzymes is expressed in nematode giant TCs [36], and a detailed histochemical study of maize BETCs [71], provides strong evidence for their lignification.

Extensins are expressed in TCs forming flange [8,11] and reticulate [18] wall ingrowths. A large number (14) of extensin family members of hydroxyproline-rich glycoproteins were expressed in developing epidermal TCs (Additional file 13), implying a key role played by extensins in shaping the ingrowth wall and, in particular, wall ingrowths. A key enzyme in the post-translational modification pathway generating functional extensins, *prolyl 4-hydroxylase* [72]*,* was expressed throughout ingrowth wall formation (Additional file 13). Upon being exocytosed into the cell wall space, extensin monomers are envisaged to cross-link to form networks, mediated by redox activity of type-III apoplasmic peroxidases ([72]; Additional file 11). Expression of a proline-rich-extensin-like receptor kinase (Additional file 3), regulating extensin/pectin interactions [73], may generate supramolecular structures [72] to function as templates for ingrowth wall formation. Significantly, coincident with wall ingrowth construction, was the expression of two nodule-specific extensins that support polarized tip growth in elongating infection threads [74] and a DZ-HRGP homolog that contributes to polarized tip growth of pollen tubes [72].

#### A large array of membrane transporters is specifically expressed in TCs

The large number of transporter genes switched off upon transiting to an epidermal TC fate (Additional file 14) suggests a substantial change in transport function.

A striking feature of the 13 TC-specific membrane transporters, whose expression did not change during *trans*-differentiation, nine transport inorganic and organic nitrogen compounds (Additional file 14). Five transport amino nitrogen compounds including two aquaglyceroporins (NIP1-2; nodulin26 – [75]; Additional file 14). Two nucleoside proton symporters, adenine/guanine permease AZG1 [76] and equilibrative nucleoside transporter 3 [77], likely function to retrieve apoplasmic nucleosides released from degradation of endosperm and inner cell layers of seed coats crushed by expanding cotyledons. Collectively, expression of these transporters reflects a demand for nitrogenous precursors to support protein biosynthesis. Expression of a putative plasma membrane potassium transporter and tonoplast potassium proton antiporter (Additional file 14) ensure potassium homeostasis.

Investment into nitrogen transporters was enhanced during uniform wall formation (Additional file 14). This included a chloride channel (CLC-7 – Additional file 14) that may function as a nitrate sensor to regulate development by modulating auxin transport [89]. Two additional transporters were recruited for potassium homeostasis; a SKOR channel (Additional file 11; [35]) and a potassium antiporter (Additional file 14). Most pronounced was the large number (12) of expressed *ABC transporter* genes (Additional file 14) linked with expression of flavonoid biosynthetic and defense genes (Table 2). However, along with effluxing xenobiotics, members of the ABCB transporter subfamily facilitate plasma membrane transport of IAA [78] contributing to the altered IAA homeostasis of epidermal TCs (see Additional file 11 and associated text).

With the onset of wall ingrowth deposition, appearance of two sugar transporter transcripts (Additional file 14) reflects a growing dependence on an extracellular carbon supply [79]. Further investments were made in amino nitrogen and potassium transporters (Additional file 14). An ongoing expression of ABC transporters (Additional file 14) corresponded with expression of flavonoid biosynthesis and defense genes (Table 2).

## Conclusions

In epidermal cells undergoing *trans*-differentiation to a TC-morphology, only a small proportion of their transcriptome (i.e., 10%) was found to be TC-specific. Within the cohort of TC-specific transcripts, those encoding pathways regulating signalling and intracellular organization were fine-tuned whilst cell wall construction and membrane transporter profiles were altered substantially upon transiting to a TC fate. Each phase of ingrowth wall construction was linked with an unique sub-cohort of expressed genes. Comparison of transcriptomes of cells forming flange or reticulate wall ingrowths detected subtle differences in their transcript profiles, with the major difference being the absence of a two-component signalling system from the latter.

### Availability of supporting data

The cDNA sequence datasets of raw reads and assembled reference transcriptome library supporting the results of this article are available in the repository of the European Nucleotide Archive (ENA) with the ENA accession number: PRJEB8906.

## Additional files

Additional file 1:
**Figure S1, Table S1 and accompanying text.** Temporal formation of the uniform wall and wall ingrowths. Additional file 2: Figure S2. Quality assessment of total RNA extracted from adaxial epidermal and storage parenchyma cells of *V. faba* cotyledons. Additional file 3: Table S2. Quality assessment of total RNA extracted from, and cDNA libraries of *V. faba* cotyledons used for Illumina sequencing. Additional file 4: Table S3. Annotation and expression levels of all genes specifically expressed in the epidermal cells of cultured *V. faba* cotyledons *trans*-differentiating to epidermal transfer cells. Additional file 5: Table S4. Annotation and expression levels (RPKM) of selected unigenes expressed specifically in *trans*-differentiating adaxial epidermal cells of *V. faba* cotyledons [80]. Additional file 6: Table S5. Primer sequences used for qRT-PCR determination of expression levels of specified genes to validate RNAseq data sets. Additional file 7: Figure S3. Species distribution of genes with highest sequence similarity to unigenes in the reference transcriptome library of *trans*-differentiating adaxial epidermal cells of cultured *V. faba* cotyledons. Additional file 8: Table S6. Reads generated from Illumina sequencing used to identify gene cohorts linked with uniform wall and wall ingrowth deposition in *trans*-differentiating epidermal cells of *V. faba* cotyledons. Additional file 9: Table S7. Temporal profile of unigene numbers expressed in adaxial epidermal or storage parenchyma cells of cultured *V. faba* cotyledons. Additional file 10:
**Figure S4 and associated text.** Cell division and expansion of adaxial epidermal cells of cultured *V. faba* cotyledons. Additional file 11: Table S8. Genes encoding proteins generating or transducing developmental signals switched off in epidermal cells transiting to a TC fate and those specifically expressed in epidermal cells undergoing *trans*-differentiation to a TC morphology. Additional file 12: Table S9. Genes encoding proteins involved in regulation of the cytoskeleton and vesicle trafficking and sphingolipid/sterol synthesis and transport that are switched off in epidermal cells transiting to a TC fate and those specifically expressed in epidermal cells undergoing *trans*-differentiation to a TC morphology. Additional file 13: Table S10. Genes encoding cell wall biosynthetic and remodelling enzymes and structural proteins switched off in epidermal cells transiting to a TC fate and those specifically expressed in epidermal cells undergoing *trans*-differentiation to a TC morphology. Additional file 14: Table S11. Genes encoding membrane transporters switched off in epidermal cells transiting to a TC fate and those specifically expressed in epidermal cells undergoing *trans*-differentiation to a TC morphology. Additional file 15: Figure S5. Validation of expression patterns of unigenes obtained from Illumina sequencing using quantitative RT-PCR.
