# Supplementary material for: Differential transcriptional networks associated with key phases of ingrowth wall construction in trans-differentiating epidermal transfer cells of Vicia faba cotyledons
Source: BMC Plant Biol. 2015 Apr 16;15:103. doi: 10.1186/s12870-015-0486-5 (PMC4437447; doi:10.1186/s12870-015-0486-5)
Supplement: Additional file 1: — Figure S1, Table S1 and accompanying text. Temporal formation of the uniform wall and wall ingrowths. [file 12870_2015_486_MOESM1_ESM.pdf]

Additional file 1:

Temporal formation of uniform wall and wall ingrowth during TC *trans*-differentiation

Methods

Epidermal peels were prepared for scanning electron microscopy to estimate percentages of epidermal cells exhibiting wall ingrowths [19]. Separate batches of cultured cotyledons were processed for transmission electron microscopy to evaluate temporal development of the uniform wall including its thickness [6].

Results and Discussion

At early times of cotyledon culture, uniform wall and wall ingrowth formation occur concurrently throughout the population of *trans*-differentiating epidermal cells (Figure S1A and B). The largest differential (4 fold) between percentages of epidermal cells engaged in these two ingrowth wall-building activities was observed at 3 h of cotyledon culture (Table S1). This differential is magnified further by the low numbers of wall ingrowths formed per cell (Figure S1A) compared to considerable deposition of uniform wall and particularly so at the corners of their juncture between the outer periclinal and anticlinal cell walls (Figure S1C). Construction of the uniform walls ceased between 8 h to 10 h of cotyledon culture (Table S1, Figure S1D). After this time, further ingrowth wall construction involved deposition of wall ingrowths alone (Table S1; [14]).

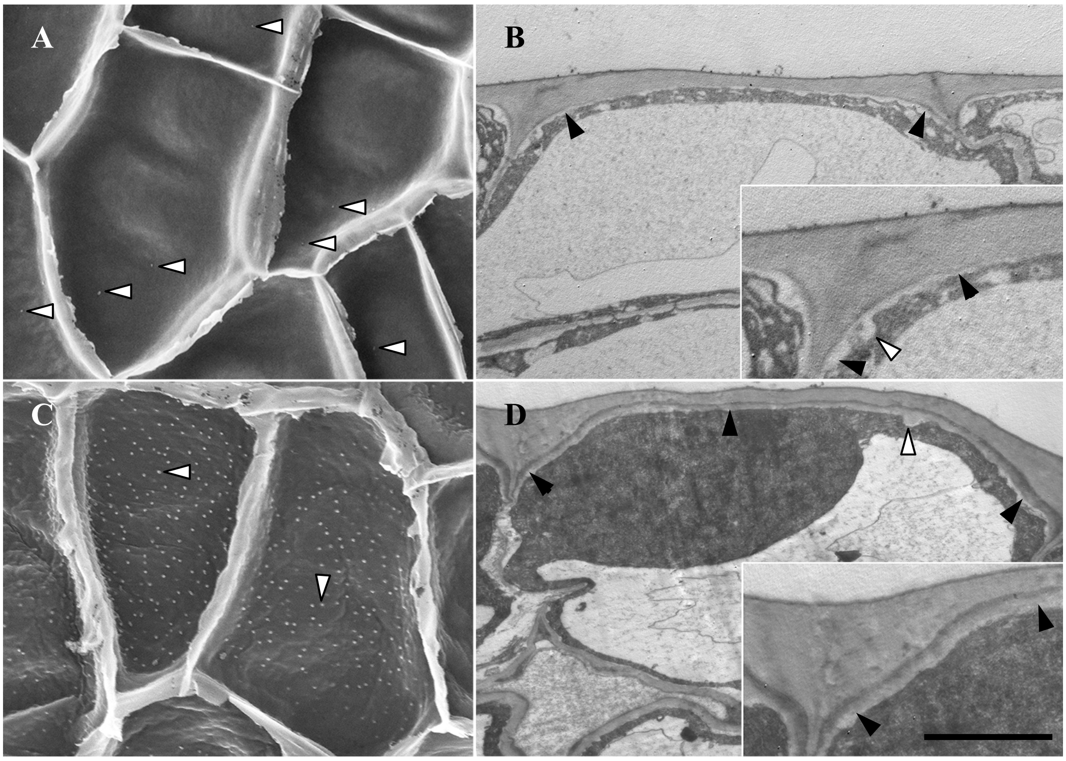

Figure S1. Electron micrograph images depicting the temporal development of the uniform wall and wall ingrowths in adaxial epidermal cells of cultured *V. faba* cotyledons. (A, C) Scanning electron microscope images of the cytoplasmic face of the outer periclinal wall of epidermal cells and (B, D) transmission electron microscope images of their transverse sections. Cotyledons were cultured for 3 h (A, B) and 12 h (C, D). An electron dense band demarcates the original wall from the uniform wall indicated by black darts in B and D. White darts with black borders indicate wall ingrowths in A to D inclusive. Bar = 20 μm for A and C; 5 μm for B and D; 2 μm for insets in B and D.

Table S1. Initiation and construction of the uniform wall and wall ingrowths in adaxial epidermal cells of cultured *Vicia faba* cotyledons. Initiation of wall formation determined as percentages of the sampled epidermal cell population exhibiting a uniform wall or wall ingrowths (see Fig. 1). Averaged estimates of uniform wall thickness as a proxy measure of its construction.

Time (h) in culture	Percent cells with:		Uniform wall thickness (nm)
	Uniform wall	Wall ingrowths	
3	42±5	10±5	103±10
10	95±3	65±8	324±10
12	96±3	73±3	318±20
