# Supplementary material for: Differential transcriptional networks associated with key phases of ingrowth wall construction in trans-differentiating epidermal transfer cells of Vicia faba cotyledons
Source: BMC Plant Biol. 2015 Apr 16;15:103. doi: 10.1186/s12870-015-0486-5 (PMC4437447; doi:10.1186/s12870-015-0486-5)
Supplement: Additional file 2: Figure S2. — Quality assessment of total RNA extracted from adaxial epidermal and storage parenchyma cells of V. faba cotyledons. [file 12870_2015_486_MOESM2_ESM.pdf]

Additional file 2:

Quality assessment of total RNA extracted from adaxial epidermal and storage parenchyma cells of *V. faba* cotyledons.

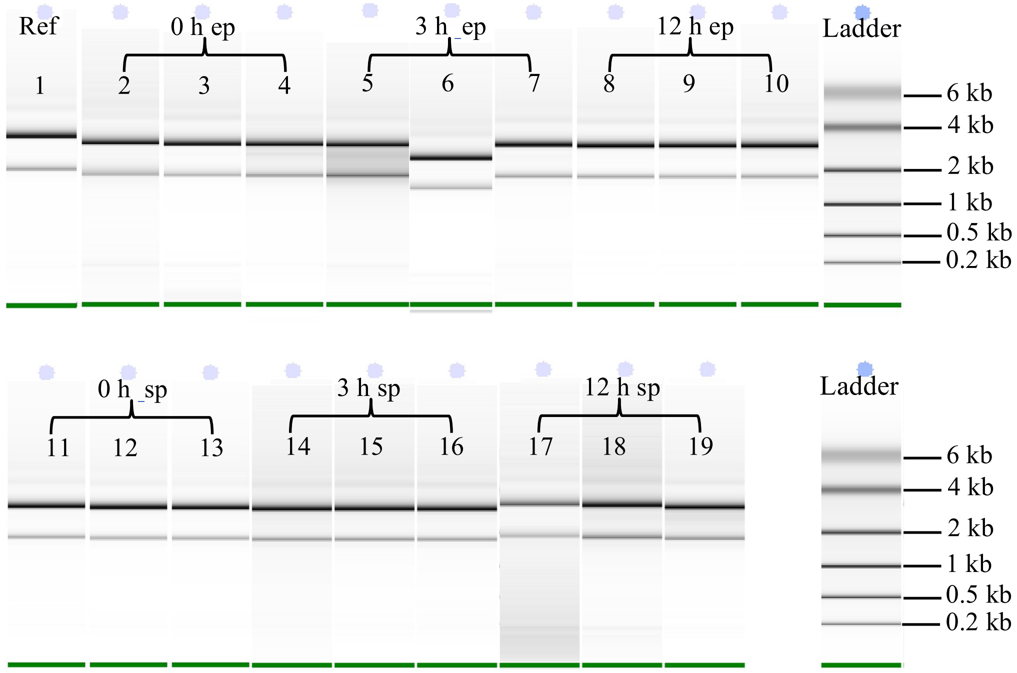

Figure S2. Quality assessment of total RNA extracted from adaxial epidermal (ep, lane 1 – 10) and storage parenchyma (sp lane 11 – 19) cells of *V. faba* cotyledons. Total RNA for construction of the *de novo* assembled reference transcriptome library (lane 1) was pooled from total RNA extracted from freshly-harvested (0 h) cotyledons and cotyledons cultured for specified times. To determine the transcriptome of *trans*-differentiating adaxial epidermal cells undergoing uniform wall or wall ingrowth deposition, total RNA was extracted from adaxial epidermal and storage parenchyma tissues of cotyledons freshly harvested (lane 2 – 4, 11 – 13) or cultured for 3 h (lane 5 – 7, 14 – 16) and 12 h (lane 8 – 10, 17 – 19).
