# Supplementary material for: Differential transcriptional networks associated with key phases of ingrowth wall construction in trans-differentiating epidermal transfer cells of Vicia faba cotyledons
Source: BMC Plant Biol. 2015 Apr 16;15:103. doi: 10.1186/s12870-015-0486-5 (PMC4437447; doi:10.1186/s12870-015-0486-5)
Supplement: Additional file 3: Table S2. — Quality assessment of total RNA extracted from, and cDNA libraries of V. faba cotyledons used for Illumina sequencing. [file 12870_2015_486_MOESM3_ESM.pdf]

Additional file 3:

Table S2. Quality assessment of total RNA extracted from, and cDNA libraries of, *V. faba* cotyledons used for Illumina sequencing. RNA was extracted from adaxial epidermal (ep) or storage parenchyma (sp) cells of cultured *V. faba* cotyledons. RNA used to construct the reference library (Reference) was pooled from total RNA extracted from freshly harvested and cultured cotyledons harvested at specified times. Transcriptomes for ep and sp were generated from tissues collected from fresh cotyledons or from 3- and 12-h cultured cotyledons.

Sample	Total RNA		cDNA library					
	Conc. (ng/µl)	RNA Integrity Number (RIN)	Calibrated conc. (ng/µl)	Molarity (nmol/l)	Peak size (bp)	Average size (bp)	Minimum size (bp)	Maximum size (bp)
Reference	111	5.6	28.2	145	254	294	178	567
0 h ep 1	422	5.2	40.2	195	249	317	171	684
0 h ep 2	204	4.8	30.8	145	259	326	178	693
0 h ep 3	146	4.7	32.4	152	254	327	178	774
0 h sp 1	142	5	31.7	153	254	319	175	665
0 h sp 2	199	4.8	32.1	159	252	310	174	667
0 h sp 3	59	5.1	30.3	155	240	301	168	650
3 h ep 1	159	4.7	32.3	156	248	318	174	706
3 h ep 2	438	4.7	22.3	99	293	347	190	711
3 h ep 3	326	4.8	28.0	133	246	325	172	690
3 h sp 1	101	4.8	32.1	150	259	329	186	558
3 h sp 2	116	4.6	25.3	119	253	325	171	675
3 h sp 3	286	4.7	29.0	135	248	329	173	730
12 h ep 1	100	4.6	30.5	145	254	323	179	717
12 h ep 2	139	5.1	41.0	185	252	347	185	817
12 h ep 3	166	4.7	31.3	152	254	317	177	691
12 h sp 1	79	7.7	29.2	144	246	310	165	671
12 h sp 2	110	9.7	28.5	127	268	345	186	776
12 h sp 3	299	4.7	17.5	76	299	355	189	730
