# Supplementary material for: Differential transcriptional networks associated with key phases of ingrowth wall construction in trans-differentiating epidermal transfer cells of Vicia faba cotyledons
Source: BMC Plant Biol. 2015 Apr 16;15:103. doi: 10.1186/s12870-015-0486-5 (PMC4437447; doi:10.1186/s12870-015-0486-5)
Supplement: Additional file 6: Table S5. — Primer sequences used for qRT-PCR determination of expression levels of specified genes to validate RNAseq data sets. [file 12870_2015_486_MOESM6_ESM.pdf]

Additional file 6:

Table S5. Primer sequences used for qRT-PCR determination of expression levels of specified genes to validate RNAseq data sets.

Cell type/IW phase	Gene ID	Forward primer	Reverse primer
Epidermal cell switched off	U11391	GCGTGGCTGTACCAACCTAT	GAGTAACGAGAAGATCGGCG
	U3705	CCTCCTCTGAATACGGCAAA	ACTTCCTAGCAAACTCCGCA
	U30408	CCCAACAAAACAAGCAGTGA	AGAACATCATTTGGAACGCC
UW/WI shared no change	U20195	ATTGCCGAAAGGATACATCG	TGAAGACACCAAAACCTCCC
	U17935	GGGCTCGTGATTCTCTACGG	AAACCACCACCCCACAAACT
	U1054	CCATCATCACAAGCACCAAC	ATTCAAATGGGGCACAACAT
UW upregulated and specific	CL2028C1	ACTGCTGCCAATGAGCTCTT	TCGAGAATTTCAGCCACGCT
	U9859	AACCGCATGCAAATCCACAC	GGTCGCTGAGGGATTGACAA
	U18006	CTCTTCCGGCGAGACAACAT	AACATTCTGAACAACCGCGC
WI upregulated and specific	U20013	CAAGGAAACAACGACGCTGG	CGTTCCCACTCTTGACGTGA
	U2598	GGCTTCCACATGACATCCCA	GTTCGGGTGTTTGGTTGTCG
	U12386	CTTAAACGCCACCATTCCGC	CTTAAACGCCACCATTCCGC
Stably expressed	CL4934C4	AGGGTTAGTGAGCACCATGC	ATAGCCAAAGGGAATACGCC
	CL7083C1	GACAACATGATTGAGAGGTCCACC	GGCTCCTTCTCAATCTCCTTACC
	CL8111C 2	GATTCTCGATGCAGGGCTAC	CCACTGCTCTTGCTCTTTCC
