# Supplementary material for: Differential transcriptional networks associated with key phases of ingrowth wall construction in trans-differentiating epidermal transfer cells of Vicia faba cotyledons
Source: BMC Plant Biol. 2015 Apr 16;15:103. doi: 10.1186/s12870-015-0486-5 (PMC4437447; doi:10.1186/s12870-015-0486-5)
Supplement: Additional file 7: Figure S3. — Species distribution of genes with highest sequence similarity to unigenes in the reference transcriptome library of trans-differentiating adaxial epidermal cells of cultured V. faba cotyledons. [file 12870_2015_486_MOESM7_ESM.pdf]

Additional file 7:

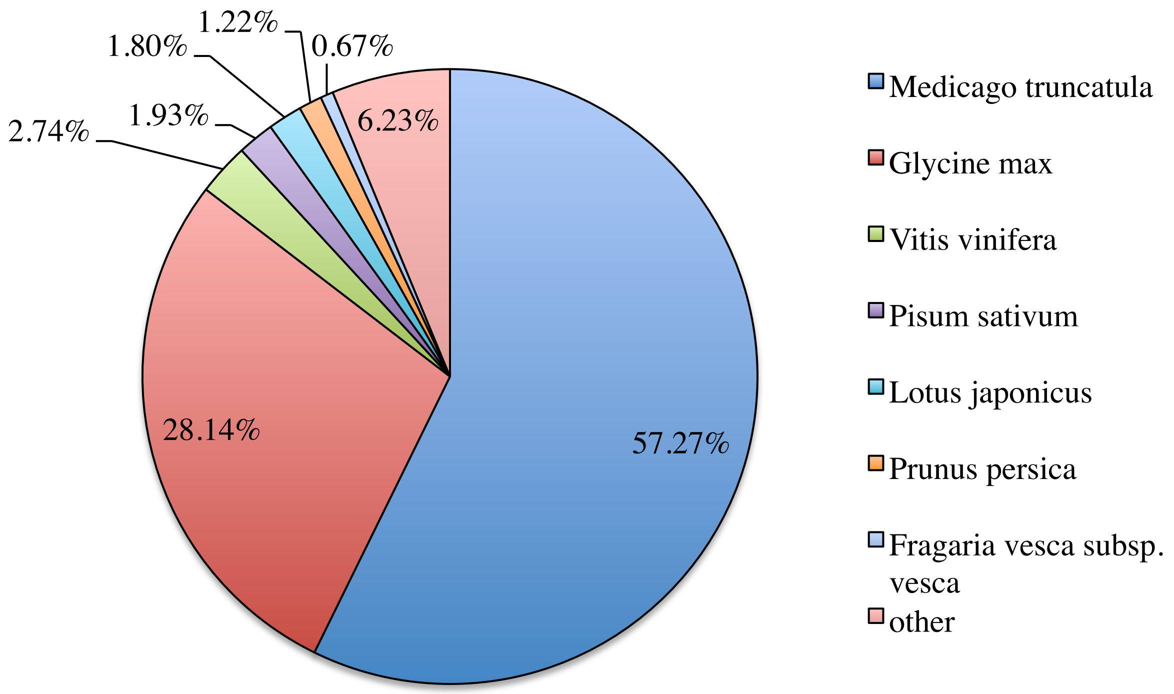

Figure S3. Species distribution of genes with highest sequence similarity (lowest BLAST E value) to unigenes in the reference transcriptome library of *trans*-differentiating adaxial epidermal cells of cultured *V. faba* cotyledons. Data obtained by aligning unigenes from the reference library with NCBI nr, Swissprot, KEGG, and COG databases using BlastX.
