# Supplementary material for: Differential transcriptional networks associated with key phases of ingrowth wall construction in trans-differentiating epidermal transfer cells of Vicia faba cotyledons
Source: BMC Plant Biol. 2015 Apr 16;15:103. doi: 10.1186/s12870-015-0486-5 (PMC4437447; doi:10.1186/s12870-015-0486-5)
Supplement: Additional file 8: Table S6. — Reads generated from Illumina sequencing used to identify gene cohorts linked with uniform wall and wall ingrowth deposition in trans-differentiating epidermal cells of V. faba cotyledons. [file 12870_2015_486_MOESM8_ESM.pdf]

Additional file 8:

Table S6. Reads generated from Illumina sequencing used to identify gene cohorts linked with uniform wall and wall ingrowth deposition in *trans*-differentiating epidermal cells of *V. faba* cotyledons. Total RNA sequenced from adaxial epidermal (ep) and storage parenchyma (sp) cells of freshly harvested (0 h) cotyledons or cotyledons cultured on MS medium for 3 and 12 h.

Sample	Raw reads	Clean reads	Mapped clean reads	% mapped clean reads	% perfect matched clean reads
0 h ep 1	37,477,731	36,400,767	31,397,825	86.26%	57.19%
0 h ep 2	25,581,177	24,860,572	21,722,916	87.38%	58.06%
0 h ep 3	26,538,570	25,825,680	22,595,495	87.49%	58.08%
0 h sp 1	30,443,345	29,396,577	24,446,740	83.16%	56.77%
0 h sp 2	38,229,224	36,998,634	31,441,909	84.98%	60.03%
0 h sp 3	36,040,869	34,805,881	28,559,365	82.05%	52.96%
3 h ep 1	30,296,809	29,426,559	25,898,156	88.01%	67.96%
3 h ep 2	34,106,713	33,096,144	28,850,639	87.17%	65.10%
3 h ep 3	29,366,660	28,397,663	24,554,446	86.47%	61.44%
3 h sp 1	32,737,030	31,794,749	26,821,369	84.36%	57.81%
3 h sp 2	30,448,510	29,423,291	24,934,716	84.74%	60.75%
3 h sp 3	34,011,695	32,987,900	27,712,021	84.01%	57.43%
12 h ep 1	37,718,811	36,695,393	32,041,507	87.32%	58.20%
12 h ep 2	32,005,465	31,132,834	26,889,958	86.37%	61.83%
12 h ep 3	23,397,571	22,639,579	19,570,804	86.45%	61.42%
12 h sp 1	19,547,655	18,601,921	15,492,084	83.28%	55.73%
12 h sp 2	31,219,651	30,067,109	24,976,404	83.07%	59.70%
12 h sp 3	23,138,669	22,192,857	18,904,761	85.18%	61.54%
