# Supplementary material for: Differential transcriptional networks associated with key phases of ingrowth wall construction in trans-differentiating epidermal transfer cells of Vicia faba cotyledons
Source: BMC Plant Biol. 2015 Apr 16;15:103. doi: 10.1186/s12870-015-0486-5 (PMC4437447; doi:10.1186/s12870-015-0486-5)
Supplement: Additional file 9: Table S7. — Temporal profile of unigene numbers expressed in adaxial epidermal or storage parenchyma cells of cultured V. faba cotyledons. [file 12870_2015_486_MOESM9_ESM.pdf]

Additional file 9:

Table S7. Temporal profile of unigene numbers expressed in adaxial epidermal or storage parenchyma cells of cultured *V. faba* cotyledons. Only unigenes with a RPKM >0.45 in all three biological replicates of each cell type for a specified sampling time were defined as being 'expressed'.

Culture time (h)	Cotyledon cell type	Unigene number
0	Adaxial epidermal cell	27,244
	Storage parenchyma cell	20,876
3	Adaxial epidermal transfer cell	29,194
	Storage parenchyma cell	23,887
12	Adaxial epidermal transfer cell	27,600
	Storage parenchyma cell	23,220
