# Supplementary material for: Differential transcriptional networks associated with key phases of ingrowth wall construction in trans-differentiating epidermal transfer cells of Vicia faba cotyledons
Source: BMC Plant Biol. 2015 Apr 16;15:103. doi: 10.1186/s12870-015-0486-5 (PMC4437447; doi:10.1186/s12870-015-0486-5)
Supplement: Additional file 10: — Figure S4 and associated text. Cell division and expansion of adaxial epidermal cells of cultured V. faba cotyledons. [file 12870_2015_486_MOESM10_ESM.pdf]

Additional file 10:

Cell division and expansion of adaxial epidermal cells of cultured *V. faba* cotyledons

Methods

Following culture of cotyledons for specified times, peels of their adaxial epidermis were prepared for microscopic examination. Mitotic indices were determined by recording frequencies of mitotic figures detected by staining with the peels with DAPI following procedures described by Dibley et al (2009). Images of cleared epidermal peels were used to derive estimates of the outer periclinal wall areas of epidermal cells using Image J software. Temporal changes in adaxial surface areas per cotyledon were obtained by capturing digital images of cotyledon adaxial surfaces at specified times throughout their culture and from these determining their areas using Image J software.

Results and Discussion

On transferring the cotyledons to culture, cell division was induced in a small population of the adaxial epidermal cells. Their mitotic indices increased to 10% at two h before declining back to basal levels by six h of cotyledon culture (Fig. S4A). Cell division activity was accompanied by a 14% increase in the mean outer periclinal wall area of each epidermal cell that plateaued at 2 h of cotyledon culture. Cell division and expansion together contributed to a remarkable 30% increase in the adaxial surface areas of cultured cotyledons that was reached by 6 h of culture (Figure S4B).

Linkage of cell division/expansion with early phases of *trans*-differentiation to a TC morphology appears to be an ubiquitous phenomenon as exemplified by BETCs of developing cereal grains (Xiong et al 2011; Thiel et al 2012a) and nematode giant TCs (Ji et al 2013). Whether this is a de-differentiation event critical for *trans*-differentiating to a TC fate remains to be determined. However, the concurrence of cell division and expansion with developmental events leading to the construction of the uniform wall need to be taken into account when interpreting the significance of gene expression profiles in relation deduced participation in building the ingrowth wall.

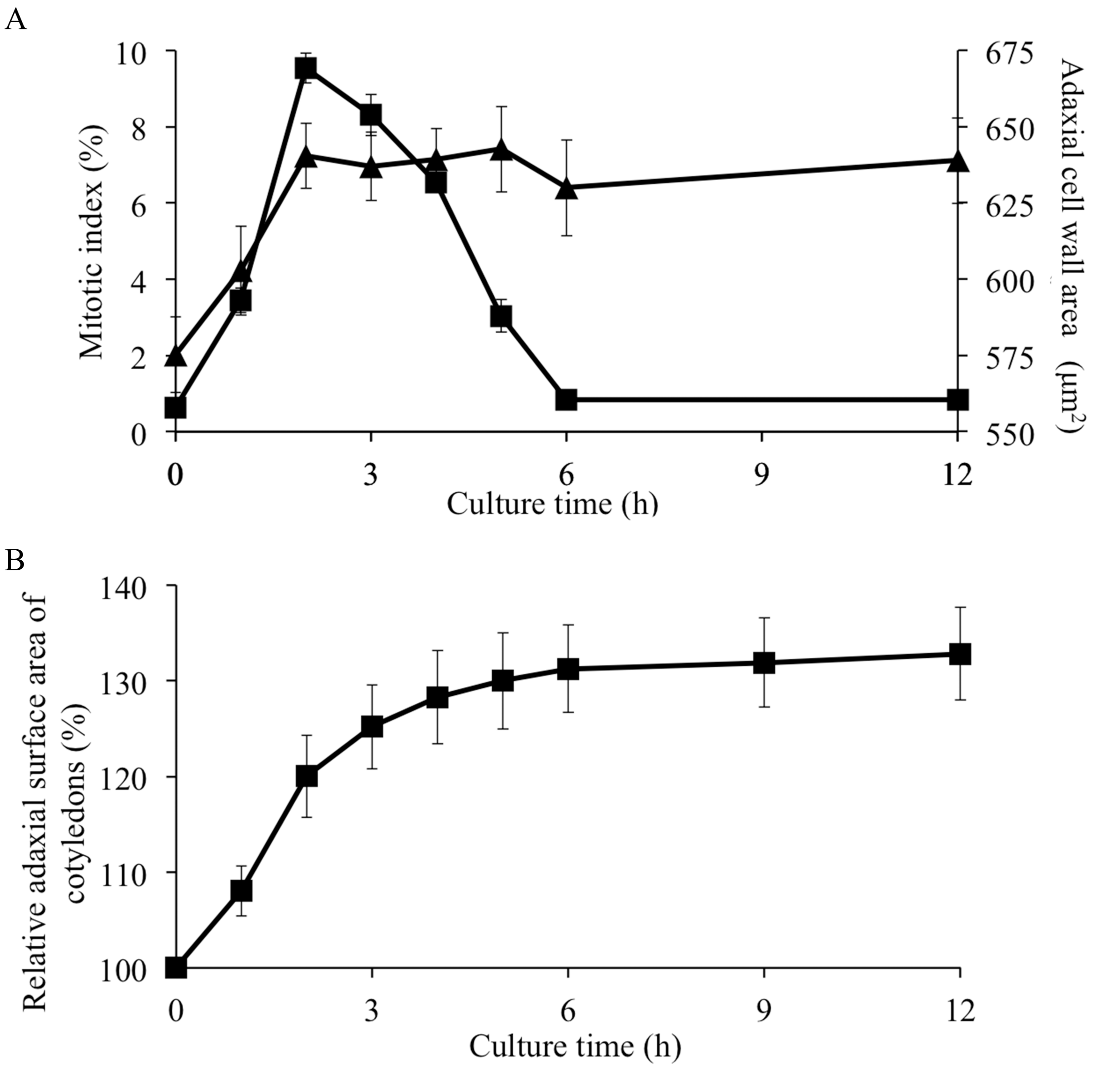

Figure S4. Temporal changes in (A) surface areas of outer periclinal walls (triangles) of, and mitotic indices (squares) in, adaxial epidermal cells of cultured *V. faba* cotyledons and (B) relative (expressed as percentages of time zero values) adaxial surface areas of cultured *V. faba* cotyledons. Cotyledons were freshly harvested or cultured on liquid MS medium for specified periods before undertaking specified measurements. (A) Mean ± SE for 200 cells per biological replicate with four replicates per time point; (B) mean ± SE of 7 replicate cultured cotyledons.
