# Supplementary material for: Differential transcriptional networks associated with key phases of ingrowth wall construction in trans-differentiating epidermal transfer cells of Vicia faba cotyledons
Source: BMC Plant Biol. 2015 Apr 16;15:103. doi: 10.1186/s12870-015-0486-5 (PMC4437447; doi:10.1186/s12870-015-0486-5)
Supplement: Additional file 11: Table S8. — Genes encoding proteins generating or transducing developmental signals switched off in epidermal cells transiting to a TC fate and those specifically expressed in epidermal cells undergoing trans-differentiation to a TC morphology. [file 12870_2015_486_MOESM11_ESM.pdf]

Additional file 11:

Table S8. Genes encoding proteins generating or transducing developmental signals switched off in epidermal cells transiting to a TC fate and those specifically expressed in epidermal cells undergoing *trans*-differentiation to a TC morphology. TC-specific genes separated into those genes expressed throughout uniform wall (UW) and wall ingrowth (WI) formation and those that are specific to each of these wall-building phases. Genes expressed throughout ingrowth wall formation are separated into groups depending on their differential expression patterns of no change, up-regulated during UW or WI formation (for more details, see Results - Transcriptome networks in epidermal cells of *in planta* and cultured cotyledons). Unigene sequences were annotated by alignment to publically available databases (see Methods) using BLASTX with an e-value threshold of $<1e^{-5}$.

Epidermal genes switched off		Transfer cell specific expressed genes									
		UW/WI no change		UW up-regulated		UW specific		WI up-regulated		WI specific	
Contig ID	Gene	Contig ID	Gene	Contig ID	Gene	Contig ID	Gene	Contig ID	Gene	Contig ID	Gene
**Auxin**											
U31687	Auxin response factor	CL3007 C9	Auxin-induced protein	U19196	AUX/IAA family member IAA4			U9020	Indole-3-acetate O-methyltransferase	CL165C 2	Indole-3-glycerol phosphate synthase
U14020	Indole-3-acetic acid inducible	CL4055 C3	Auxin-induced protein like protein					CL2550 C1	Auxin efflux carrier protein	CL7523 C1	Auxin influx protein
								U8726	Auxin-induced protein	U17577	Auxin-induced protein 6B
								CL989 C4	PCNT115	U6397	Class III HD-Zip protein 2
										U33780	MYB TF/ KAN
										U3299	WUSCHEL-related homeobox 5 (WOX5)
										U3298	WUSCHEL
										U37422	SAUR
										U3409	RAMOSUS5
**Ethylene**											
U11391	Ethylene receptor	CL3488 C2	1-aminocyclopropane-1-carboxylate oxidase	CL8157 C2	Aminocyclopropane-1-carboxylic acid synthase	CL2028 C1	1-aminocyclopropane-1-carboxylate synthase	U8572	1-aminocyclopropane-1-carboxylate oxidase	U15922	Aminocyclopropane-1-carboxylate oxidase
		U18687	Ethylene-responsive transcription factor RAP2-6	U225	ERF019	CL2028 C2	1-aminocyclopropane-1-carboxylate synthase 7			U12540	Ethylene responsive transcription factor 1a
						U18915	ERF110				
						U18987	ERF110				
						U14736	ERF110				
						U24481	ERF054-like				
						U30633	ERF SHINE				
**Gibberellins**											
CL1664 C1	Transcription factor GAMYB	U20036	Short internode related sequence	U30186	Gibberellin 2-beta-dioxygenase 8-like	CL5309 C2	Gibberellin 20 oxidase 1-like				
						CL449C 1	Gibberellin 3-beta-dioxygenase 4-like				
						U25483	2-beta-dioxygenase 2				
						U16470	Gibberellin 2-oxidase				

**Abscisic acid**											
CL4429 C4	type I inositol-1,4,5-trisphosphate 5-phosphatase CVP2-like	U17050	PYL6	U32247	Transcription factor LEC1-A	U16482	protein phosphatase 2C 25-like	CL5144 C1	ABR18	U20017	Abscisic acid hydroxylase 2-like
		CL5483 C2	Mps one binder kinase activator-like 1A			U15900	Transcription factor bHLH122|			U15674	Aldehyde oxidase 3
						U21705	WRKY transcription factor 33-like				
						U16630	WRKY transcription factor 40-like				
**Cytokinins**											
				U25276	Cytokinin-O-glucosyltransferase	U35673	Cytokinin-O-glucosyltransferase	U23225	Cytokinin dehydrogenase	CL458 C2	Cytokinin-O-glucosyltransferase
				U7541	Cytokinin-O-glucosyltransferase	U28147	Cytokinin-O-glucosyltransferase				
**ROS**											
U10474	Nucleobase-ascorbate transporter 11-like			U21465	L-ascorbate oxidase	U2649	L-gulonolactone oxidase-like	CL8648 C2	Peroxidase	U6799	Respiratory burst oxidase homolog protein E-like
U22639	Peroxidase 19-like									U37639	Carboxymethylenebutenolidase-like protein
										CL8648 C1	Peroxidase
**Calcium**											
		U23903	Cyclic nucleotide-gated channel	U23902	Cyclic nucleotide-gated channel	Unigene 29153	Autoinhibited calcium ATPase			U36148	IQ-DOMAIN 1-like containing protein
		U18508	Annexin	U29950	Cyclic nucleotide-gated channel 15 like	U20796	Calcium-dependent protein kinase 1-like			U11156	IQ domain-containing protein
		U35348	IQ-DOMAIN 14–like protein	CL4204 Cl	Potassium channel SKOR	U20795	Calcium-dependent protein kinase 2-like				
				CL1526 C1	Type IIB calcium ATPase MCA5	U20217	Calcium-dependent protein kinase 24-like				
				U18269	Calcium-binding protein CML16-like	U20889	Copine03				
				U14697	Calcium binding protein						
				U12681	Guanylyl cyclase						
				U984	MLO1						
