# Supplementary material for: Differential transcriptional networks associated with key phases of ingrowth wall construction in trans-differentiating epidermal transfer cells of Vicia faba cotyledons
Source: BMC Plant Biol. 2015 Apr 16;15:103. doi: 10.1186/s12870-015-0486-5 (PMC4437447; doi:10.1186/s12870-015-0486-5)
Supplement: Additional file 12: Table S9. — Genes encoding proteins involved in regulation of the cytoskeleton and vesicle trafficking and sphingolipid/sterol synthesis and transport that are switched off in epidermal cells transiting to a TC fate and those specifically expressed in epidermal cells undergoing trans-differentiation to a TC morphology. [file 12870_2015_486_MOESM12_ESM.pdf]

Additional file 12:

Table S9. Genes encoding proteins involved in regulation of the cytoskeleton and vesicle trafficking and sphingolipid/sterol synthesis and transport that are switched off in epidermal cells transiting to a TC fate and those specifically expressed in epidermal cells undergoing *trans*-differentiation to a TC morphology. TC -specific genes are separated into genes expressed throughout uniform wall (UW) and wall ingrowth (WI) formation and those that are specific to each of these wall-building phases. Genes expressed throughout ingrowth wall formation are separated into groups depending on their differential expression patterns of no change, up-regulated during UW or WI formation (for more details, see – Results, Transcriptome networks in epidermal cells of *in planta* and cultured cotyledons). Unigene sequences were annotated by alignment to publically available databases (see Methods) using BLASTX with an e-value threshold of $<1e^{-5}$.

**Epidermal switched off**		**Transfer cell specific expressed genes:**									
		**UW/WI no change**		**UW upregulated**		**UW specific**		**WI upregulated**		**WI specific**	
Contig ID	Gene	Contig ID	Gene	Contig ID	Gene	Contig ID	Gene	Contig ID	Gene	Contig ID	Gene
**Cytoskeleton and vesicle trafficking**											
U22353	Kinesin-4	U1553	Similar to gb|U06698 neuronal kinesin heavy chain from Homo sapiens and contains a PF|00225 Kinesin motor domain. EST gb|AA042507 comes from this gene [Arabidopsis thaliana]			CL2210 C1	actin			CL2130 C1	65-kDa microtubule-associated protein 3-like [Glycine max] MAP65-5 in Arabidopsis
U10534	Kinesin-like protein	U1054	Formin-like protein			U8030	actin-97-like			U16493	Kinesin-like polypeptide
CL6198 C2	125 kDa kinesin-related protein-like					CL6200 C1	Myosin XI			CL7887 C2	Villin-4
U36967	formin-like protein 8-like										
U11691	Protein ABIL1										
U3882	Myosin-like protein										
CL6120 C6	Rab-GDP dissociation inhibitor	CL6109 C2	GTP-binding protein SAR1A	U18006	ADP-ribosylation factor GTPase-activating protein AGD12	U21725	ADP-ribosylation factor GTPase-activating protein	U9470	syntaxin-112-like	U12467	ADP-ribosylation factor GTPase-activating protein AGD14-like isoform X1
U15938	GTPase obg	CL4945 C3	ER-derived vesicles protein ERV14	U12722	Dynamin-related protein 1C-like isoform 2	U4133	Rab-GDP dissociation inhibitor			CL4811 C2	Exocyst complex component SEC3A-like

CL1125 C1	small GTP-binding protein, partial	U19005	signal peptide peptidase-like 2B-like			U35180	Brefeldin A-inhibited guanine nucleotide-exchange protein			U9471	Syntaxin-112
U5119	Probable exocyst complex component 6-like	U7624	Plant synaptotagmin [			U21554	Exocyst complex component			CL3098 C3	Dynamin-2B-like
						U5838	Endoplasmic oxidoreductin-1				
						U9284	Vesicle-associated protein 2-1-like				
						U2172	Vesicle-associated membrane protein				
**Sphingolipid and sterol biosynthesis and transport**											
		CL2667 C2	3-ketoacyl-CoA synthase	U6945	Biotin carboxyl carrier protein of acetyl-CoA carboxylase	U32221	Oxysterol-binding protein-related protein	U12386	Epoxide hydrolase	U11800	3-ketoacyl-CoA synthase 21-like
		U7625	non-specific lipid-transfer protein AKCS9-like	CL6235C 1	3-ketoacyl-CoA synthase	CL10929 C1	Long-chain-fatty-acid-CoA ligase			U35890	Elongation of fatty acids protein
		CL9118C 2	bax inhibitor 1-like	CL5837C 1	3-ketoacyl-CoA synthase	CL4043	Remorin			U31293	Squalene epoxidase
				U7915	beta-ketoacyl-CoA synthase					U14071	Epoxide hydrolase
				U8918	non-specific lipid-transfer protein AKCS9-like					U40880	lipid transfer protein precursor
										U12302	PCTP-like protein-like
										U32592	Long-chain-alcohol O-fatty-acyltransferase
