# Supplementary material for: Differential transcriptional networks associated with key phases of ingrowth wall construction in trans-differentiating epidermal transfer cells of Vicia faba cotyledons
Source: BMC Plant Biol. 2015 Apr 16;15:103. doi: 10.1186/s12870-015-0486-5 (PMC4437447; doi:10.1186/s12870-015-0486-5)
Supplement: Additional file 13: Table S10. — Genes encoding cell wall biosynthetic and remodelling enzymes and structural proteins switched off in epidermal cells transiting to a TC fate and those specifically expressed in epidermal cells undergoing trans-differentiation to a TC morphology. [file 12870_2015_486_MOESM13_ESM.pdf]

Additional file 13:

Table S10. Genes encoding cell wall biosynthetic and remodelling enzymes and structural proteins switched off in epidermal cells transiting to a TC fate and those specifically expressed in epidermal cells undergoing *trans*-differentiation to a TC morphology. TC-specific genes are separated into those genes expressed throughout uniform wall (UW) and wall ingrowth (WI) formation and those that are specific to each of these wall-building phases. Genes expressed throughout ingrowth wall formation are separated into groups depending on their differential expression patterns of no change, up-regulated during UW or WI formation (for more details, see – Results, Transcriptome networks in epidermal cells of *in planta* and cultured cotyledons). Unigene sequences were annotated by alignment to publically available databases (see Methods) using BLASTX with an e-value threshold of <1e^{-5}.

Epidermal cell genes switched off		Transfer-cell specific expressed genes									
		UW/WI no change		UW up-regulated		UW specific		WI up-regulated		WI specific	
Contig ID	Gene	Contig ID	Gene	Contig ID	Gene	Contig ID	Gene	Contig ID	Gene	Contig ID	Gene
**Cellulose**											
U3705	Cellulose synthase catalytic subunit			U35532	GPI-anchored protein	U31167	Endoglucanase	U30949	Acidic endochitinase-like protein	CL1800.C5	Beta-glucosidase
U15597	Cellulose synthase catalytic subunit					CL3775.C2	Endoglucanase				
CL7904 C1	Cellulose synthase-like protein H1					U30633	ERF SHINE				
**Matrix polysaccharides**											
CL9188.C1	Xylan 1 4-beta-xylosidase	CL8806C2	Xylosyltransferase	U15114	Beta-D-xylosidase 5-like	CL8806.C2	Xylosyltransferase	CL6667.C1	Beta-galactosidase	U34223	Alpha-D-xylosidase
U1567	Expansin	CL10235.C1	Expansin-A4-like	CL6394.C1	Xyloglucan-specific endoglucanase inhibitor protein	U3247	Glycosyltransferase family GT8 protein	U41126	Subtilisin-like protease	CL8194.C2	Alpha-xylosidase-like
U30965	Galacturonosyl-transferase 11	CL2572.C3	Alpha-expansin	U15415	NAC domain protein NAC2	U3248	Glycosyltransferase family GT8 protein	U26002	Glucan endo-1,3-beta-glucosidase	U12075	Alpha-expansin
U24012	Pectinesterase	CL9884.C2	Pectinesterase	U9859	Pectinesterase/ pectinesterase inhibitor 41-like	U22861	Xyloglucan endotransglycosylase hydrolase 2			U4017	Beta-galactosidase
U31151	Pectinesterase			CL5918.C1	Polygalacturonase inhibiting protein	CL9193.C1	Xyloglucan endotransglucosylase/hydrolase			U36200	UDP-glucuronosyl-transferase 1-1
U5021	Pectinesterase			U20328	Cinnamoyl CoA reductase	U31050	Xyloglucan endotransglucosylase/hydrolase			U20013	Endo-beta-1 3-glucanase
U2932	Subtilisin-like serine protease					U8264	Xyloglucan endotransglucosylase/hydrolase			U16698	Trans-cinnamate 4-monooxygenase
U38413	Callose synthase					CL7192.C3	Xyloglucan endotransglucosylase				
U30408	Glucan endo-1,3-beta-d-glucosidase					U16662	Expansin-A12-like				

						CL4158. C2	Galacturonosyltransferase 13-like				
						CL7790. C1	Pectinesterase				
						CL7192. C!	Pectinesterase				
						U29365	N-hydroxycinnamoyl /benzoyltransferase				
						CL9121. C2	Cinnamyl alcohol dehydrogenase-related protein				
**Cell wall structural proteins**											
U10288	Fasciclin-like arabinogalactan protein	CL5752. C2	Prolyl 4-hydroxylase subunit alpha-1	U26015	Extensin class 1 protein	U13038	Arabinogalactan peptide	CL6100. C3	Extensin nodule-specific proline-rich protein	CL6100.C 2	Extensin=nodule-specific proline-rich protein
U22417	Fasciclin-like arabinogalactan protein	U9245	Extensin	U901	Extensin-like protein	U34866	TPA: putative proline-rich extensin-like receptor protein kinase family protein	CL6100. C3	Extensin=nodule-specific proline-rich protein	U11743	extensin=nodule-specific proline-rich protein
		CL7881. C1	Glycine-rich protein	U14236	Extensin-like protein	U274	Putative glycine rich protein precursor	CL5023. C1	Hydroxyproline-rich glycoprotein DZ-HRGP	U8399	Pollen Ole e 1 allergen and extensin
								CL5788. C1	Repetitive proline-rich cell wall protein	CL5788.C 2	Repetitive proline-rich cell wall protein
								CL3930. C1	Pherophorin-S	CL8701.C 2	Stem 28 kDa glycoprotein
