# Supplementary material for: Differential transcriptional networks associated with key phases of ingrowth wall construction in trans-differentiating epidermal transfer cells of Vicia faba cotyledons
Source: BMC Plant Biol. 2015 Apr 16;15:103. doi: 10.1186/s12870-015-0486-5 (PMC4437447; doi:10.1186/s12870-015-0486-5)
Supplement: Additional file 14: Table S11. — Genes encoding membrane transporters switched off in epidermal cells transiting to a TC fate and those specifically expressed in epidermal cells undergoing trans-differentiation to a TC morphology. [file 12870_2015_486_MOESM14_ESM.pdf]

Additional file 14:

Table S11. Genes encoding membrane transporters switched off in epidermal cells transiting to a TC fate and those specifically expressed in epidermal cells undergoing *trans*-differentiation to a TC morphology. TC-specific genes separated into those genes expressed throughout uniform wall (UW) and wall ingrowth (WI) formation and those that are specific to each of these wall-building phases. Genes expressed throughout ingrowth wall formation are separated into groups depending on their differential expression patterns of no change, up-regulated during UW or WI formation (for more details, see – Results, Transcriptome networks in epidermal cells of *in planta* and cultured cotyledons). Unigene sequences were annotated by alignment to publically available databases (see Methods) using BLASTX with an e-value threshold of <1e^{-5}.

**Epidermal cell genes switched off**		**Transfer cell specific expressed genes**									
		**UW/WI shared**		**UW up-regulated**		**UW specific**		**WI up-regulated**		**WI specific**	
Contig ID	Gene	Contig ID	Gene	Contig ID	Gene	Contig ID	Gene	Contig ID	Gene	Contig ID	Gene
U33031	Bidirectional sugar transporter SWEET4-like	CL7004C1	Amino acid permease AAP4	CL7082C1	Amino-acid permease,	U18246	Sugar transporter	U2598	ABC transporter G family	CL2123C2	Hexose transporter
U20727	Mannitol transporter	CL8772.C1	Peptide/nitrate transporter	CL5269C1	Proline transporter	U40718	Cl-channel clc-7			U4242	Carbohydrate transporter
U13817	Metal transporter CNNM4	CL2654C2	Sodium-coupled neutral amino acid transporter	U16412	Ammonium transporter 1 member	U12068	K(+)/H(+) antiporter			U4615	Peptide transporter PTR3-B
U33928	Zn/Cd P(IB)-type ATPase	U24787	High affinity nitrate transporter 2.5-like	U8713	Inorganic phosphate transporter 1-4	U11616	Sulfate transporter			CL8772.C1	Peptide/nitrate transporter
U10474	Equilibrative nucleoside transporter 3-like	U17935	Ammonium transporter 3	U19486	Zn/Cd P(IB)-type ATPase	U3485	ABC transporter B family member			U38725	Potassium channel AKT1-like
U15675	Equilibrative nucleoside transporter 3-like	U19937	Adenine/guanine permease AZG1-like	CL5406C1	Metal transporter CNNM4	U29103	Multidrug resistance protein ABC transporter family,			U36661	SST1-like protein
U36010	TRANSPARENT TESTA 12-like	CL703C7	Equilibrative nucleoside transporter 3-like	CL734C1	PREDICTED: pleiotropic drug resistance protein 1-like	U29110	ABC transporter C family member 10-like			U40089	Magnesium-transporting ATPase, P-type 1-like
U25453	ABC transporter	CL8954C2	Aquaporin NIP1-2	U29110	ABC transporter C family member 10-like	U4110	ABC transporter C family member 9-like			U5521	ABC transporter C family member 10-like
U1457	ABC transporter B family member	U20977	Nodulin26-like intrinsic protein	U16609	ABC transporter C family member 9-like	U3473	TRANSPARENT TESTA			U2872	ABC transporter
U10945	ABC transporter G	U19332	K(+)/H(+) antiporter	U22590	Multidrug and toxin extrusion protein (MATE)	U3010	MATE efflux family protein DTX1-like			U36776	ABC transporter
U3778	Ripening regulated protein DDTFR18	CL10233C2	Potassium transporter	U29612	Ripening regulated protein DDTFR18	U35103	Ripening regulated protein DDTFR18			U5686	TRANSPARENT TESTA
U32217	Multidrug resistance protein ABC transporter	U24874	ABC transporter I			U17657	Zinc finger protein STOP1 homolog			U539	Heavy metal transport/detoxification protein
		U3474	TRANSPARENT TESTA							U29622	Metal tolerance protein 10-like
