# Supplementary material for: Differential transcriptional networks associated with key phases of ingrowth wall construction in trans-differentiating epidermal transfer cells of Vicia faba cotyledons
Source: BMC Plant Biol. 2015 Apr 16;15:103. doi: 10.1186/s12870-015-0486-5 (PMC4437447; doi:10.1186/s12870-015-0486-5)
Supplement: Additional file 15: Figure S5. — Validation of expression patterns of unigenes obtained from Illumina sequencing using quantitative RT-PCR. [file 12870_2015_486_MOESM15_ESM.pdf]

Additional file 15:

Validation of expression patterns of unigenes obtained from Illumina sequencing using quantitative RT-PCR.

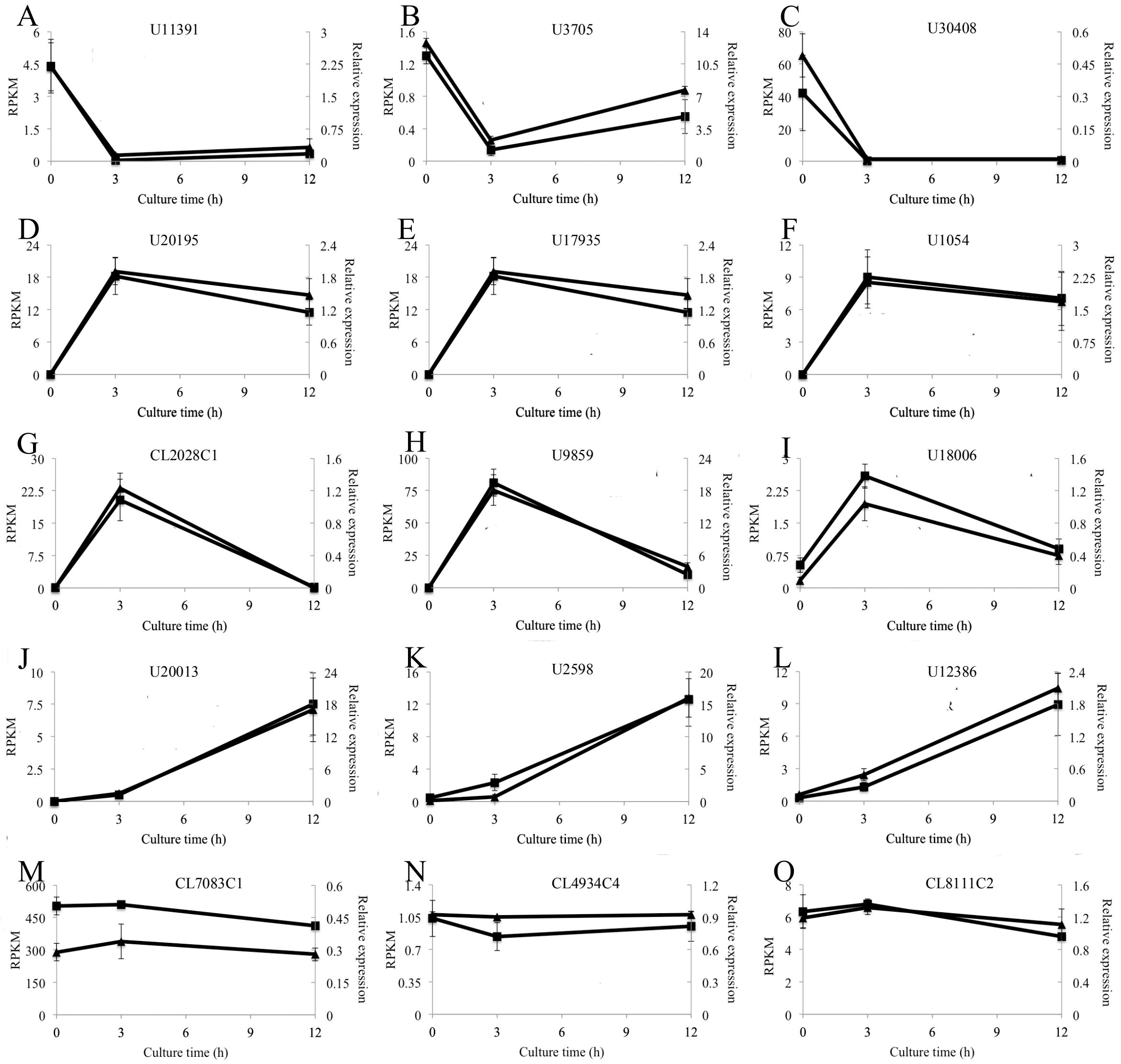

Figure S5. Validation of expression patterns of unigenes obtained from Illumina sequencing using quantitative RT-PCR. Fifteen genes exhibiting five expression patterns were analyzed. These were: (A – C) epidermal cell switched off; (D – F) uniform wall/wall ingrowth shared no change; (G – I) uniform wall up-regulated; (J – K) wall ingrowth up-regulated; (M – O) stably expressed. Temporal changes in RPKM value (closed squares) and qRT-PCR expression levels (closed triangles) for each selected unigene are presented. U11391, ethylene receptor; U3705, cellulose synthase catalytic subunit; U 30408, glucan endo-1,3-beta-d-glucosidase; U20195, anthranilate N-benzoyltransferase; U17935, ammonium transporter 3 member; U 1054, formin-like protein; CL2028C1, 1-aminocyclopropane-1-carboxypectinesterase inhibitor 41-like synthase; U9859, pectinesterase inhibitor 41-like; U18006, ADP-ribosylation factor GTPase-activating protein AGD12; U20013, endo-beta-1, 3-glucanase; U2598, ABC transporter G family member 11-like; U12386, epoxide hydrolase; CL7083C1, elongation factor 2 alpha; CL4934C4, NADH dehydrogenase subunit 4; CL8111C2, 60S ribosomal protein L2. For all qRT-PCR reactions, U9834 (multidomain cyclophilin type peptidyl-prolyl cis-trans isomerase) was used as the reference gene. All data are mean ± SE from three biological replicates.
